# Design, Synthesis, and Anticancer Activity of Novel 3,6-Diunsaturated 2,5-Diketopiperazines

**DOI:** 10.3390/md21060325

**Published:** 2023-05-26

**Authors:** Xiaolin Li, Tianrong Xun, Huayan Xu, Xiaoyan Pang, Bin Yang, Junfeng Wang, Xuefeng Zhou, Xiuping Lin, Suiyi Tan, Yonghong Liu, Shengrong Liao

**Affiliations:** 1 Research Center for Marine Microbes, CAS Key Laboratory of Tropical Marine Bio-Resources and Ecology, Guangdong Key Laboratory of Marine Materia Medica, South China Sea Institute of Oceanology, Chinese Academy of Sciences, Guangzhou 510301, China; lixiaolin211@mails.ucas.ac.cn (X.L.); xypang@scsio.ac.cn (X.P.); yangbin@scsio.ac.cn (B.Y.); wangjunfeng@scsio.ac.cn (J.W.); xfzhou@scsio.ac.cn (X.Z.); xiupinglin@hotmail.com (X.L.); 2 University of Chinese Academy of Sciences, Beijing 100049, China; 3 Department of Pharmacy, Southern Medical University, Shenzhen 518100, China; xtr21200791@smu.edu.cn; 4 Wuya College of Innovation, Shenyang Pharmaceutical University, Shenyang 110016, China; xhy6013@126.com; 5 NMPA Key Laboratory for Research and Evaluation of Drug Metabolism, Guangdong Provincial Key Laboratory of New Drug Screening, School of Pharmaceutical Sciences, Southern Medical University, Guangzhou 510515, China

**Keywords:** 2,5-diketopiperazine derivative, intermolecular hydrogen bond, liposolubility, electron property, anticancer activity

## Abstract

Based on the marine natural products piperafizine B, XR334, and our previously reported compound **4m**, fourteen novel 3,6-diunsaturated 2,5-diketopiperazine (2,5-DKP) derivatives (**1**, **2**, **4**–**6**, **8**–**16**), together with two known ones (**3** and **7**), were designed and synthesized as anticancer agents against the A549 and Hela cell lines. The MTT assay results showed that the derivatives **6**, **8**–**12,** and **14** had moderate to good anticancer capacities, with IC_50_ values ranging from 0.7 to 8.9 μM. Among them, compound **11,** with naphthalen-1-ylmethylene and 2-methoxybenzylidene functions at the 3 and 6 positions of 2,5-DKP ring, respectively, displayed good inhibitory activities toward both A549 (IC_50_ = 1.2 μM) and Hela (IC_50_ = 0.7 μM) cancer cells. It could also induce apoptosis and obviously block cell cycle progression in the G2/M phases in both cells at 1.0 μM. The electron-withdrawing functions might not be favorable for the derivatives with high anticancer activities. Additionally, compared to piperafizine B and XR334, these semi-*N*-alkylated derivatives have high liposolubilities (>1.0 mg mL^−1^). Compound **11** can be further developed, aiming at the discovery of a novel anticancer candidate.

## 1. Introduction

Cancer has become a main threat to human health and life around the world [1]. The migration, resistance, and occurrence of cancer cells cause treatment failure even when modern techniques, such as surgery, radiation, chemotherapy, immunotherapy, or their combination, are employed. Fighting against cancer is a hard and time-consuming war, and new approaches are needed urgently, in which developing novel anticancer agents with high potency still plays a critical role in fighting against this disease [2].

**Figure 1:**
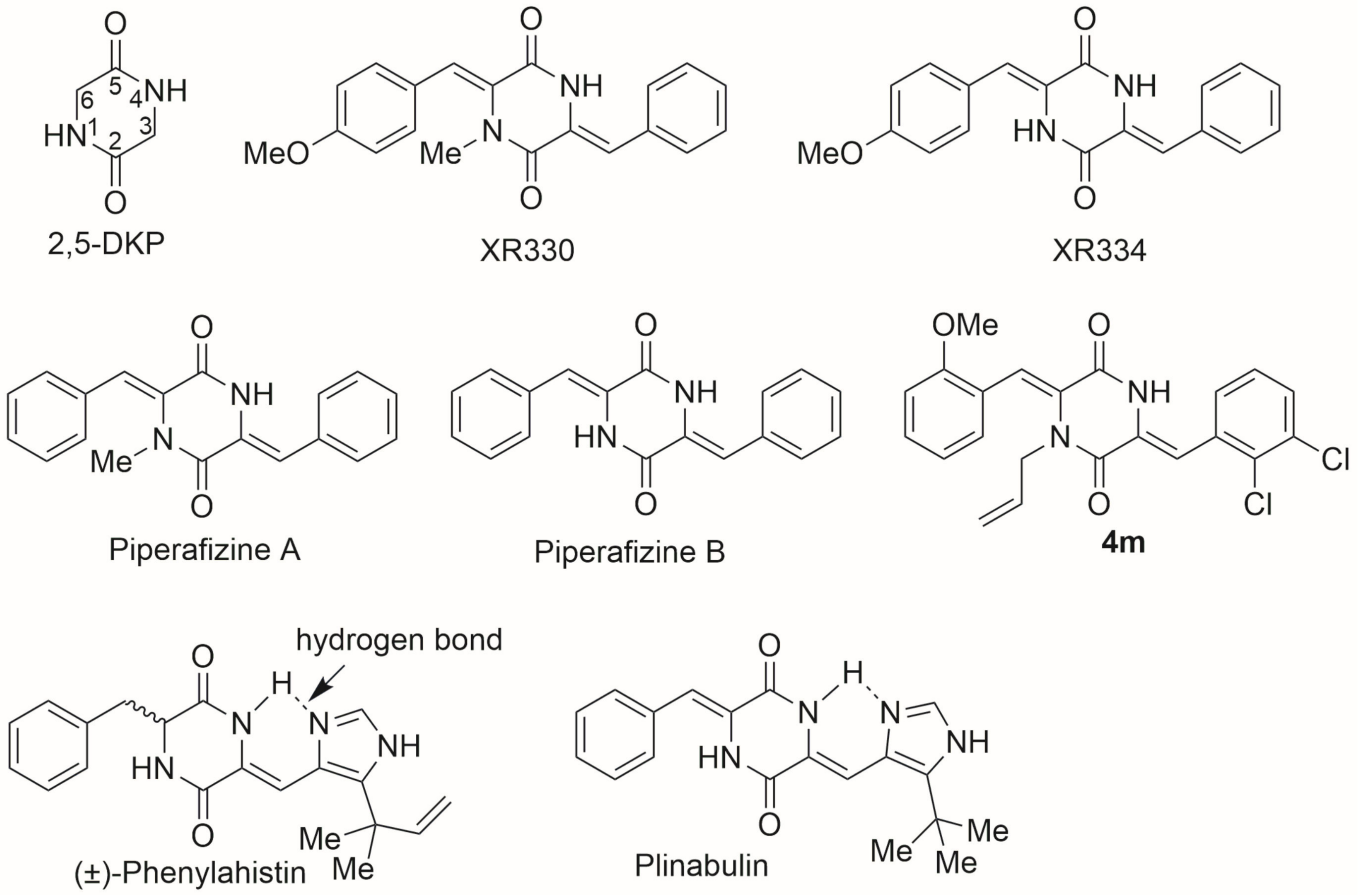
Structure of 2,5-DKP and its representative 2,5-DKPs with 3,6-diunsaturated C–C double bonds.

**Figure 2:**
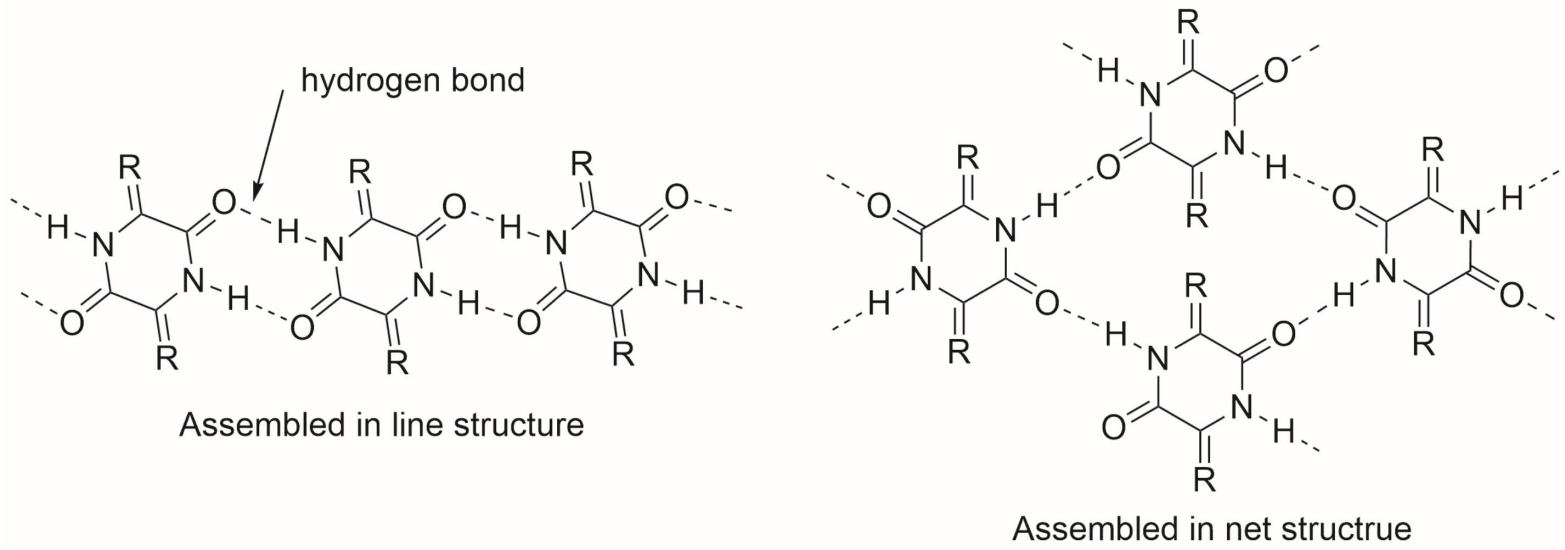
2,5-DKPs with 3,6-diunsaturated C–C double bonds assembled in line or net structure.

2,5-diketopiperazine (2,5-DKP) is the smallest cyclic peptide and a useful scaffold frequently found in numerous structurally diverse natural products [3,4,5]. 2,5-Diketopiperazine derivatives (2,5-DKPs) have higher stability than their linear counterparts against enzymolysis [6] and often have conformational rigidity and are able to interact with various biological targets [7], thus giving rise to a broad range of biological activities [5,8], such as antivirus [9], anticancer [10,11,12,13,14], antifouling [15,16], antioxidation [17], and anti-PAI-1 [18,19] activities. As a result, 2,5-DKP has become an attractive and privileged scaffold for the discovery of highly active pharmaceutical agents. One class of 2,5-DKPs, which have unsaturated C–C double bonds at the 3 and/or 6 positions of the 2,5-DKP ring, such as the marine natural products XR334 [18], piperafizine B [20], (±)-phenylahistin [21], and its synthetic derivative plinabulin [22], possess two pairs of hydrogen bond donors and acceptors (Figure 1) and show markedly different anticancer properties. The 2,5-DKPs furnished with phenyl rings at the 3 and 6 positions, such as piperafizine B and XR334, assemble in line and/or net frameworks due to the formation of the intermolecular hydrogen bond as well as the π–π stacking interactions [5,13] (Figure 2). These compounds have very poor liposolubilities and weak anticancer activities, and further biological investigations into them are thus prevented [19]. However, those 2,5-DKPs with an imidazole (or 2-pyridyl) group as a side component, such as (±)-phenylahistin and plinabulin, have an intramolecular hydrogen bond formed preferentially between the amide hydrogen of the 2,5-DKP ring and the nitrogen atom of the side imidazole moiety [23] (Figure 1), and the tendency for the formation of the intermolecular hydrogen bonds is largely weakened. Their liposolubilities and anticancer activities are greatly increased [14]. The lipophilicities of this kind of compound can also be improved by the introduction of protective groups, similar to the semi-*N*-methylation of piperafizine B to A or XR334 to XR330, on the amide nitrogen atom of a 2,5-DKP ring. Previous studies demonstrated that these semi-*N*-protected 2,5-DKPs had improved liposolubilities and could cross the cell membrane easily, along with enhanced stable capacities away from the degradation caused by enzymes [6]. Furthermore, the enhanced lipophilicity of 2,5-DKPs was beneficial to their anticancer activity [24].

**Scheme 1:**
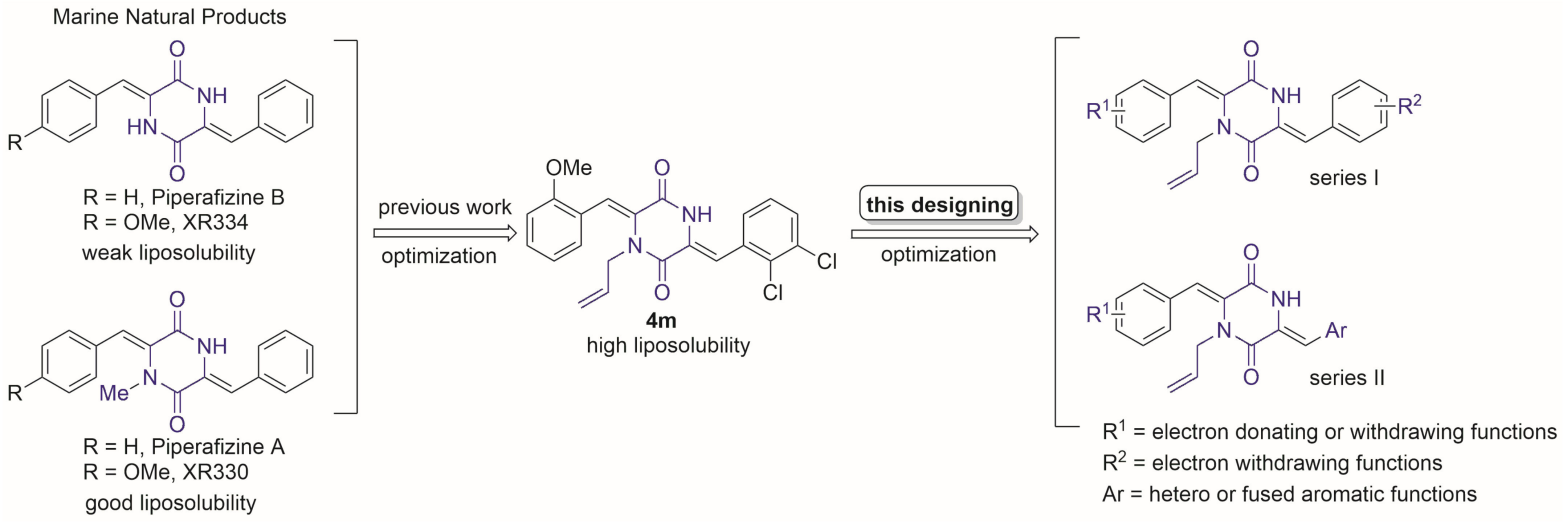
Designing of the 2,5-DKPs in this study.

In our previous study, compound **4m** showed good anticancer activities against several cell lines, including the A549 and Hela cell lines [13]. An allyl group was introduced to the 1-nitrogen atom of the 2,5-DKP ring to interrupt the formation of the intermolecular hydrogen bonds. Compared to the methyl group, this function with a little longer side chain could also disturb the π–π stacking interactions between the layers of the frameworks, which then contribute to the good lipophilicities of the derivatives. On the other hand, the biological results showed that the 2-methoxy group (2-OMe) on the phenyl ring at the 6 position of the 2,5-DKP ring played a critical role in improving the anticancer activity, whereas the impacts of its two chlorine atoms and some other strong electron-withdrawing groups (e.g., NO_2_, CF_3_, and CN) on the anticancer activities of derivatives have not been clearly investigated, as electron properties sometimes exhibit significant (positive or negative) impacts on a derivative’s bioactivity [25,26]. Similarly, the size or skeleton of the substituent will also have different influences on the anticancer activities of derivatives [7,27]. Therefore, based on the marine natural products piperafizine B and XR334, in this context, a small library of novel 2,5-DKPs was designed as follows (Scheme 1): (1) similar to compound **4m**, the allyl group on the 1-nitrogen atom was retained; (2) the electron-withdrawing groups, such as NO_2_, CN, CF_3_, Br, Cl, and F on the phenyl group (series I), and the electron donating group OMe on benzene (series I or II) or naphthalene (series II) were introduced; and (3) a few electron-rich (e.g., furan and thiophene), electron-deficient (e.g., pyridine) heteroaromatic scaffolds, or fused naphthyl or quinolyl functions (series II) were employed. Thus, fourteen novel 2,5-DKPs (**1**, **2**, **4**–**6**, **8**–**16**), along with two known ones (**3** and **7**), were designed and synthesized, and their anticancer activities against two cell lines, A549 and Hela, were evaluated aiming at the discovery of highly active anticancer agents.

## 2. Results and Discussion

### 2.1. Synthesis of Compound

**Scheme 2:**
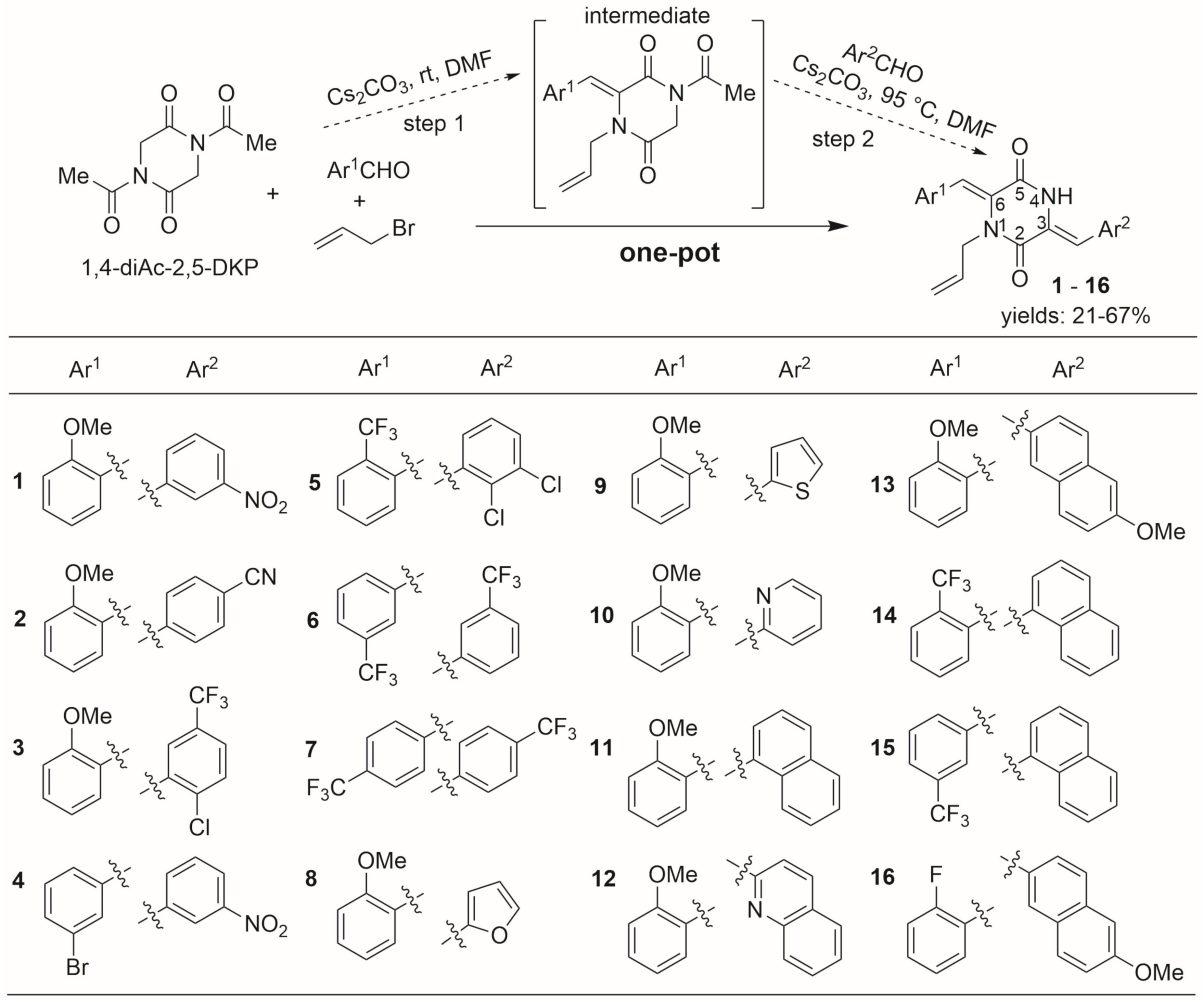
Synthesis of derivatives **1**–**16**.

By following our previously reported procedure [28], the compounds were synthesized using a one-pot operation (Scheme 2). For the synthesis of compounds **1**–**5** and **8**–**16**, in the first step, after the reaction of 1,4-diAc-2,5-diketopiperazine with the corresponding aldehyde and allyl bromide under base conditions at room temperature to yield the intermediate, another corresponding aldehyde was added directly in the second step, and the reaction was subsequently heated under base conditions until the disappearance of the intermediate. Of note is that the base additive used in two steps can be added together in the first step. The syntheses of compounds **6** and **7** were similar to the above procedure, whereas the corresponding aldehydes were added just once at the first step. The products have low to moderate yields ranging from 21 to 67%.

**Figure 3:**
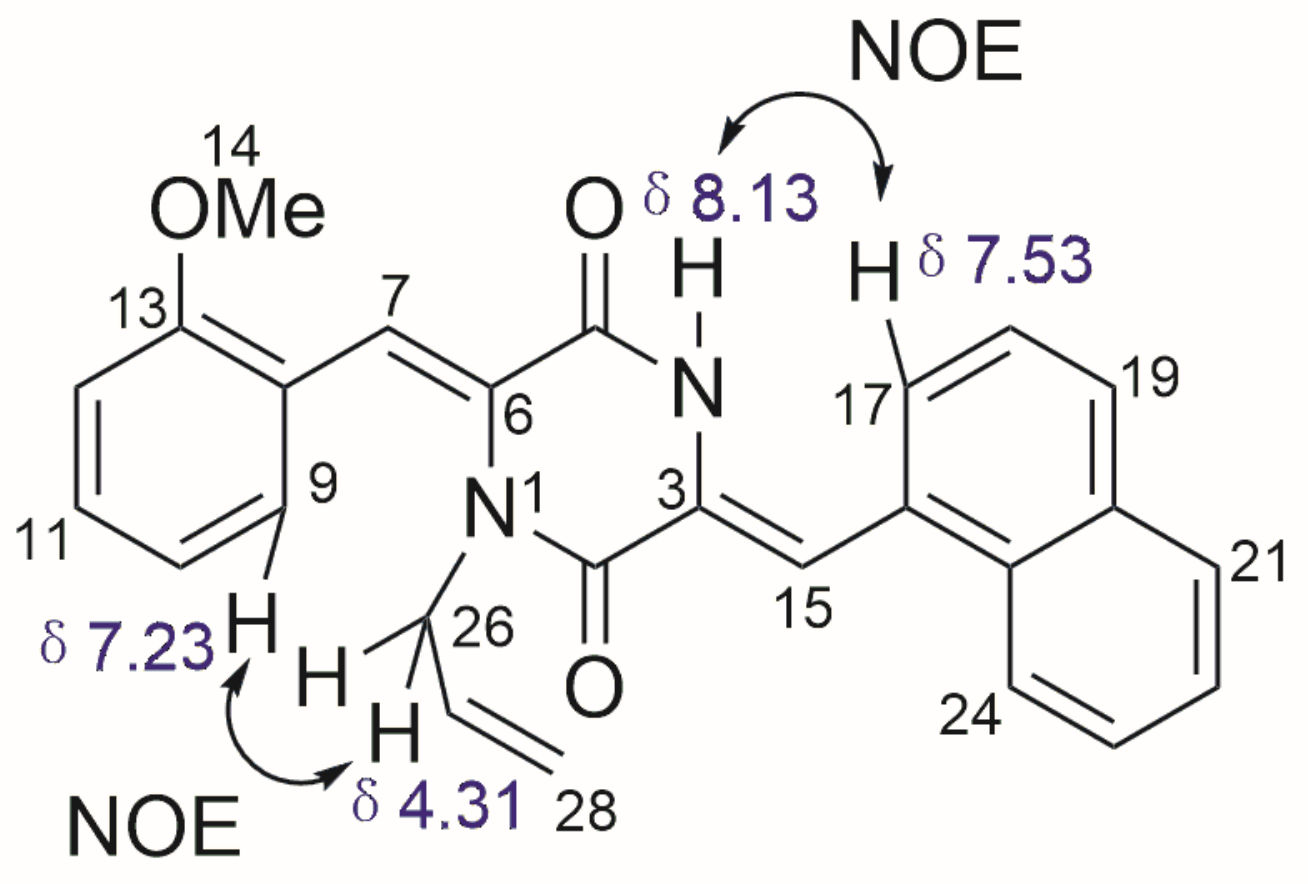
Characterization of compound **11**.

Compound **11** was selected as a representative compound to verify the correct structure of this kind of product based on its NMR and HRMS results (Figure 3). The ^1^H NMR spectrum displayed that there are eleven aromatic proton signals (*δ*_H_ 8.01–8.00, H-24; 7.88–7.86, H-21; 7.85, H-19; 7.56–7.50, H-23, H-22, H-17, H-18; 7.37–7.35, H-11; 7.25, H-9; 7.00, H-10; 6.93, H-12). Note that one alkene terminal proton signal is overlapped (*δ*_H_ 7.56–7.50, H-15), and the other alkene terminal proton signal is at 7.30 for H-7. The *δ*_H_ values ranging from 5.61 to 4.31 are signals of allyl, and the signal of the methoxyl group is at 3.87. The ^13^C NMR spectrum showed that two amide carbon signals (*δ*_C_ 159.8 and 158.9) and one aromatic carbon (C-13, adjacent to the methoxyl group) signal (*δ*_C_ 157.3) are in the far low-field. There are twenty-one carbons signals (*δ*_C_ 133.8–110.6), which belong to the groups of naphthalene (10 carbons), benzene (5 carbons), and three C–C double bonds (6 carbons). The HRMS (ESI) result confirmed the correct molecular formula C_26_H_23_N_2_O_3_ (*m/z* 411.1696 [M + H]^+^) for this compound. The 2D NMR (NOESY) spectrum displayed that the ^1^H–^1^H correlations occurred between the H-9 (*δ*_H_ 7.23) and H-26 (*δ*_H_ 4.31), H-4 (*δ*_H_ 8.13) and H-17 (*δ*_H_ 7.53) bonds (please see Figure S29 in Supplementary Materials), which indicates the correct stereo-configuration for the *3Z*,*6Z*-isomer and is similar to those in the previous studies [12,28].

**Scheme 3:**
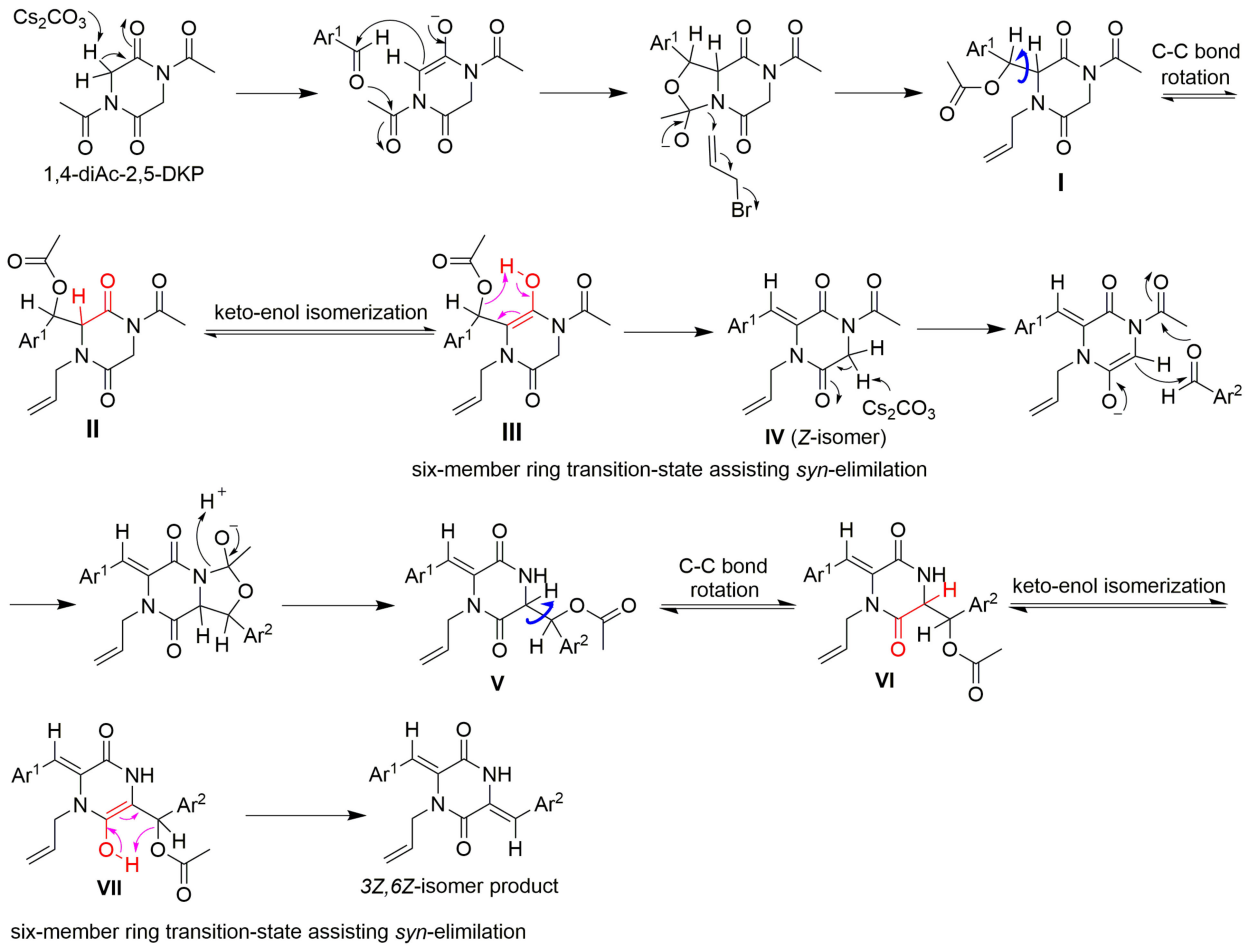
Proposed mechanism for the selective formation of *3Z*,*6Z*-2,5-DKPs.

**Scheme 4:**
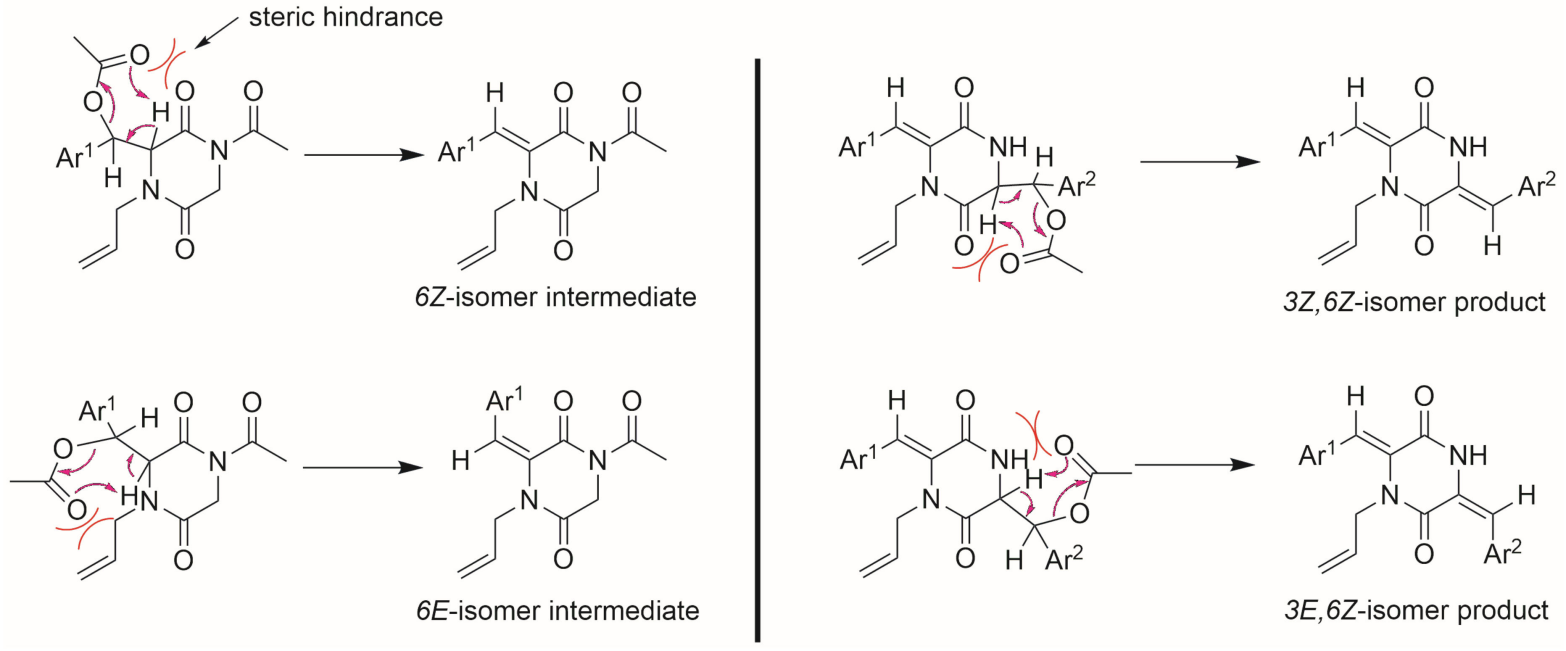
Isomers formed via Chugaev-like elimination might be prevented due to steric hindrance.

A proposed mechanism for the selective formation of this kind of product with a *3Z*,*6Z* configuration is as follows (Scheme 3). Firstly, 1,4-diAc-2,5-DKP reacts with Ar^1^CHO to afford the key intermediate **I**, which then forms intermediate **II** via the C–C bond rotation. Through a keto-enol isomerization, **II** will become intermediate **III** and subsequently yield intermediate **IV** (*Z*-isomer) via a *syn*-elimilation promoted by an intramolecular six-member ring transition state. Next, **IV** reacts with Ar^2^CHO to give intermediate **V**, and this key intermediate forms the final *3Z*,*6Z*-2,5-DKPs via a sequential transformation similar to those following intermediate **I**. Surely, the formation of 6*Z* or 6*E* intermediates and *3Z*,*6Z*- or *3E*,*6Z*-2,5-DKPs following a Chugaev-like elimination might be prevented due to steric hindrance (Scheme 4).

The solubility test demonstrated that all the compounds could be soluble in common organic solvents, such as dimethyl sulfoxide (DMSO), *N*,*N*-dimethylformamide (DMF), ethyl acetate (AcOEt), dichloromethane (DCM), chloroform, and acetone, with the solubilities > 1.0 mg mL^−1^ at room temperature.

### 2.2. Biological Evaluation of Derivatives

#### 2.2.1. Inhibiting Proliferation of Cancer Cells

First, we screened the antiproliferative effects of all compounds against the cancer cell lines A549 and Hela at a preliminary concentration of 10.0 μM. Those with inhibition >50% were selected for further detailed evaluations, and their IC_50_ values were subsequently calculated and obtained (Table 1). It was found that compounds **8**–**11** showed moderate to good inhibitory capacities against cell line A549 (IC_50_ = 1.2–7.3 μM), while compounds **6**, **8**–**12**, and **14** showed moderate to good activities against the Hela cell line (IC_50_ = 0.7–8.9 μM). It seemed that the active compounds had a little stronger anticancer activity against Hela than A549. In comparison to compound **4m**, the antiproliferative activities of the active compounds were not obviously improved, except for compound **11**, which had nearly 1.9-fold activity (IC_50_ = 1.2 μM) stronger than compound **4m** (IC_50_ = 2.3 μM) against the A549 cell line and around 2.3-fold activity (IC_50_ = 0.7 μM) stronger than compound **4m** (IC_50_ = 1.6 μM) against the Hela cell line. The results showed that the compounds with electron-withdrawing groups, such as 3-NO_2_ (**1**), 4-CN (**2**) or 2-Cl-5-CF_3_ (**3**), on the phenyl group at the 3 position of the 2,5-DKP ring had weak activities (IC_50_ > 10.0 μM). Similar to our previous study, when replacing 2-OMe with 3-Br (**4**), 2-CF_3_ (**5**), 3-CF_3_ (**6**) or 4-CF_3_ (**7**) on the phenyl group at the 6 position of the 2,5-DKP ring, only compound **6** had moderate inhibitory activity against Hela cells (IC_50_ = 8.9 μM). Of note is that these compounds also contain electron-withdrawing groups, including 3-NO_2_ (**4**), 2,3-Cl (**5**), 3-CF_3_ (**6**) or 4-CF_3_ (**7**) on the phenyl group at the 3 position of the 2,5-DKP ring. These results imply that electron-withdrawing groups might be not suitable functions. Delightedly, when using electron-rich heteroaromatic cycles such as furan (**8**) and thiophene (**9**) or electron-deficient pyridine (**10**) as the substituents on the 3 position of the 2,5-DKP ring, compounds had moderate to good activities against both the A549 (IC_50_ = 3.7–7.3 μM) and Hela (IC_50_ = 4.7–5.9 μM) cell lines. Changing the substituent of 1-naphthalene (**11**) into 2-quinoline (**12**) or 6-methoxy-2-naphthalene (**13**), compound **12** displayed only moderate activity against Hela cells (IC_50_ = 6.2 μM), while compound **13** showed no obvious activities toward either of the cancer cell lines. Surprisingly, compound **14**, by taking the place of the 2-OMe with 2-CF_3_ on the phenyl group at the 6 position of the 2,5-DKP ring, could also suppress the growth of the Hela cell line with good activity (IC_50_ = 3.9 μM). However, by moving the 2-CF_3_ from the 2 position to the 3 position (**15**) or replacing 2-OMe with 2-F (**16**), the activities of the compounds were all lost. Collectively, in terms of the above results, although compound **14** with a 2-CF_3_ moiety shows good activity, other examples (compounds **1**–**7**) indicate that the electron-withdrawing functions on phenyl groups at both sites (3 and 6 positions) of the 2,5-DKP ring might be not favorable. In addition to the vital role of the 2-OMe group, the substitutive motifs with suitable sizes or skeletons at the 3 position of the 2,5-DKP ring might be another critical factor for the 2,5-DKPs with high anticancer activities. It should be mentioned that multiple factors, such as the skeleton, size, type, and/or position of the substituent, might have combined impacts on the anticancer properties of these derivatives.

**Figure 4:**
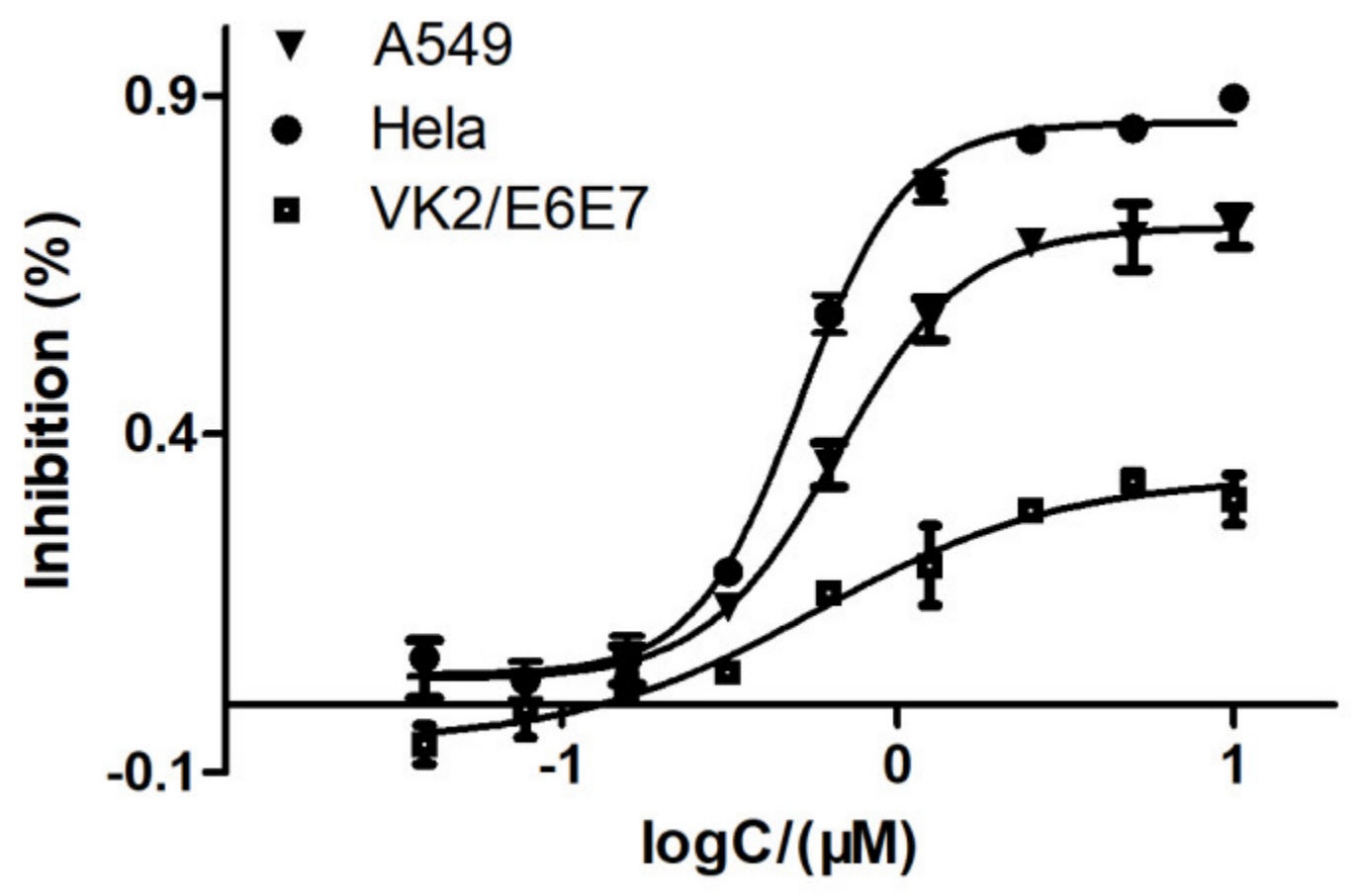
Relationships between the inhibition and the concentration of compound **11** for two cancer cell lines (A549 and Hela) and a normal cell line (VK2/E6E7). All results are presented as mean ± standard deviation (SD) from three parallel experiments.

The results also showed that compound **11** could inhibit the growth of both cell lines in dose-dependent manners after treatment for 48 h, and this compound had a slightly stronger inhibitory activity against Hela than A549 (Figure 4). In addition, the cytotoxicity of compound **11** on human ectocervical–vaginal epithelial cells (VK2/E6E7 cells) was examined, and the results showed that it had low toxicity to normal cells (only 30% inhibition at 10.0 μM), implying its value for further optimization.

#### 2.2.2. Apoptosis in Cancer Cell Lines Induced by Compound **11**

**Figure 5:**
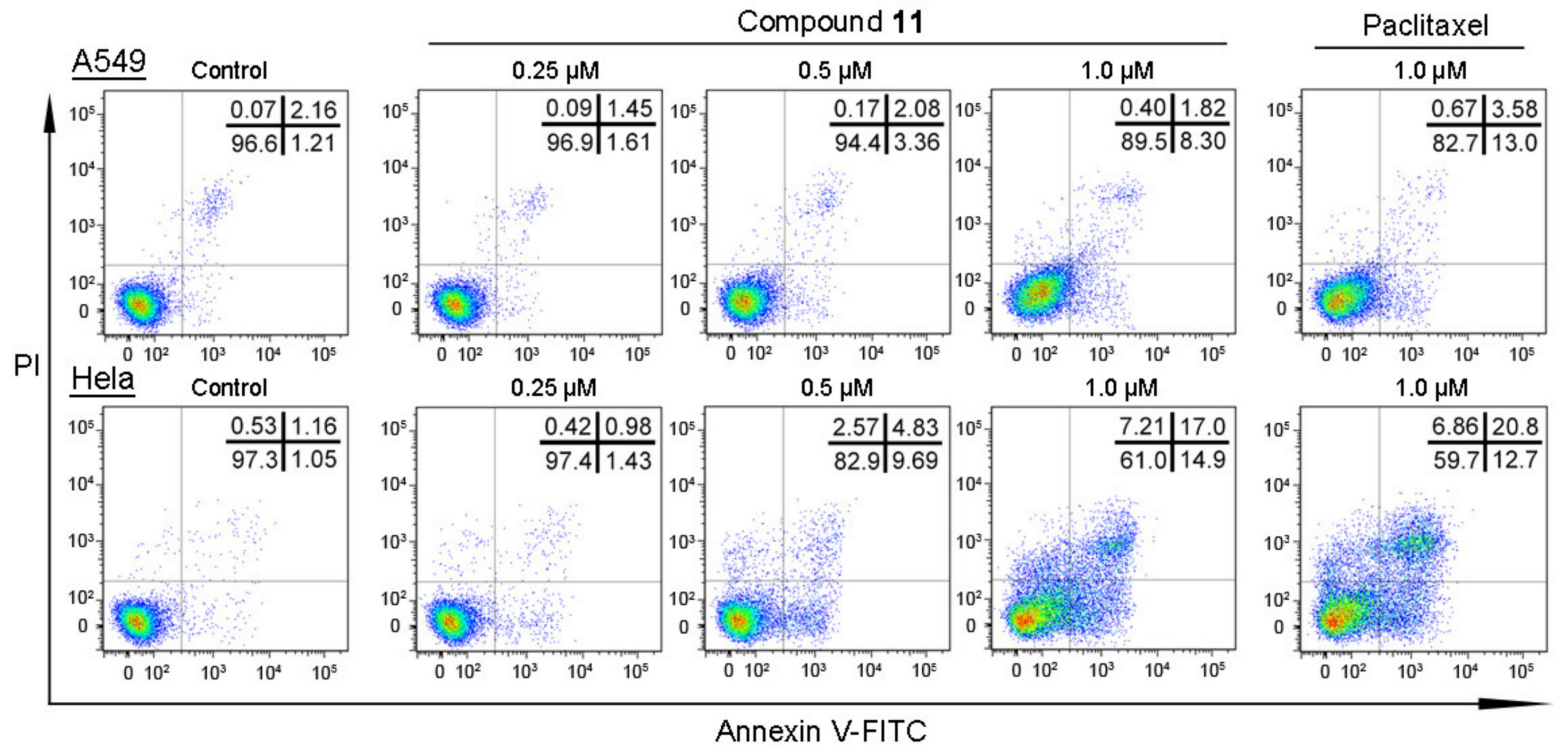

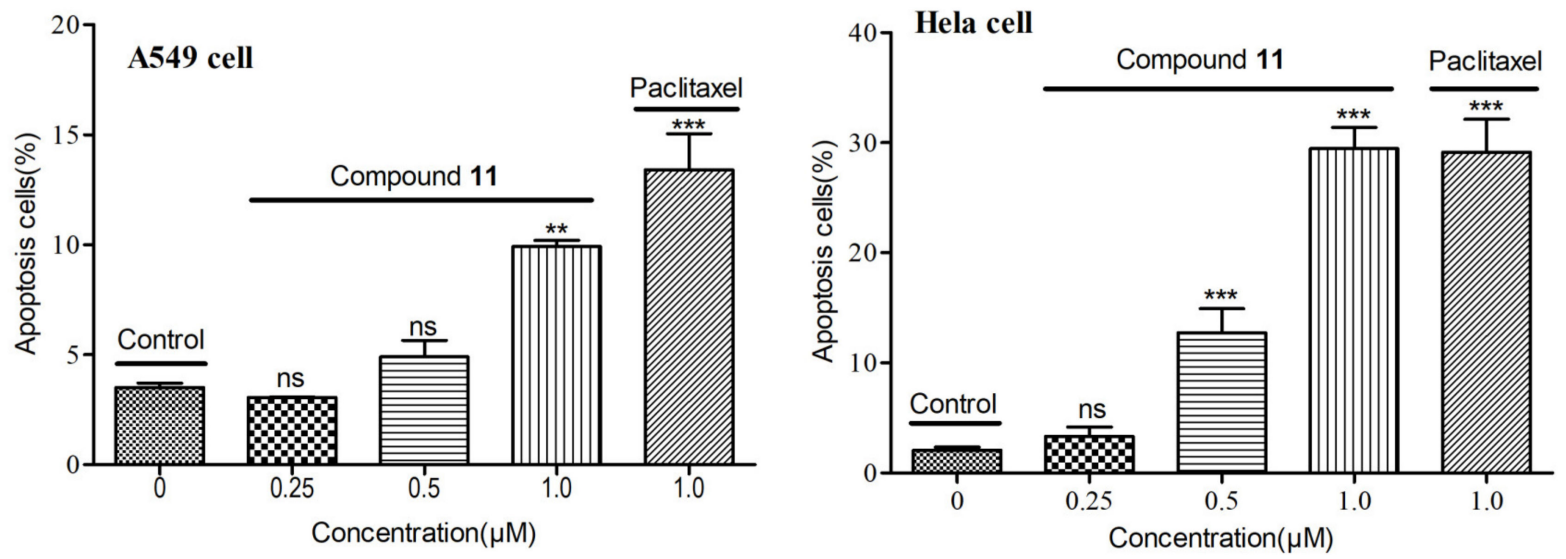
Compound **11** induced apoptosis in A549 and Hela cancer cells. PBS (pH 7.4) containing DMSO (1‰) was used as a negative control, and paclitaxel was used as a positive control. All results were presented as mean ± standard deviation (SD). “ns” means no statistical differences; ** *p* < 0.01, *** *p* < 0.001, Student’s *t*-test.

Previous studies demonstrated that 3,6-diunsaturated 2,5-DKPs with anticancer properties showed a phenotype characteristic of inducing apoptosis in cancer cells. For example, the synthetic plinabulin with a side imidazole component could induce apoptosis in multiple myeloma cells [29]. Our former synthetic compound **3c** with lipidic side alkyl chain could also induce apoptosis in U937 cells with dose- and time-dependent relationships [30]. Therefore, the abilities of compound **11** to induce apoptosis in both cell lines were evaluated using flow cytometry with Annexin V-FITC and PI dual staining. As shown in Figure 5, compound **11** could obviously induce apoptosis in both cells at 1.0 μM after 48 h of treatment in comparison with the untreated controls. Note that cell death occurred in both early and late stages. Dose-dependent effects of compound **11** on the induction of apoptosis in both cells were observed, especially for Hela cells, indicating that Hela cells were more sensitive to this compound, which is consistent with the MTT results that compound **11** had a stronger inhibitory ability against Hela than A549. Surprisingly, necrosis characterized by a positive correlation between the amount and the treated concentrations of compound **11** in Hela cells was also found, implying this compound might simultaneously work through other unknown pathways to induce cell death, and similar results were also found in a former study that focused on a deuterated plinabulin [31].

#### 2.2.3. Cell Cycle Arrest Induced by Compound **11**

**Figure 6:**
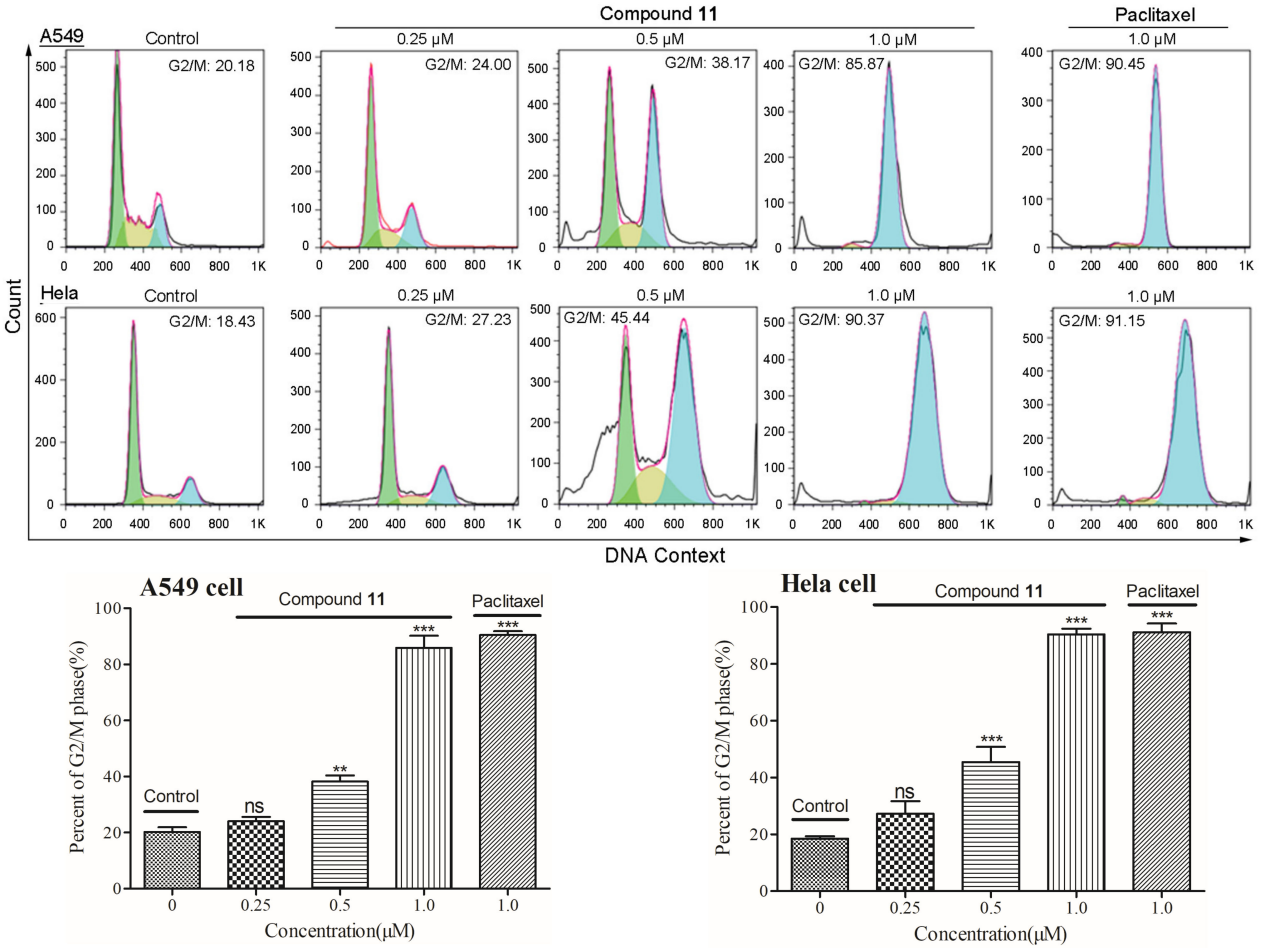
Compound **11** significantly induced G2/M phase arrest in both cancer cell lines compared to control. PBS (pH 7.4) containing DMSO (1‰) was used as a negative control, and paclitaxel was used as a positive control. All results were presented as mean ± standard deviation (SD). “ns” means no statistical differences; ** *p* < 0.01, *** *p* < 0.001, Student’s *t*-test.

Cell cycle arrest experiments were also performed to investigate which phase of the cell cycle progression was blocked by compound **11**. As shown in Figure 6, compound **11** could arrest both cancer cells in the G2/M phases, and the cell contents increased following the concentration gradients. Compound **11** also showed a stronger potency to arrest the Hela cells than A549 cells, consistent with the above MTT and apoptosis results. Compared to the untreated controls, major peak distributions for the G2/M phases could be found when cells were treated with 0.5 μM, and the cell cycle progression could be almost blocked at the G2/M phase with percentages of 85.87 (A549) and 90.37% (Hela), respectively, when treated with 1.0 μM for 24 h. The results were similar to the cell cycles blocked by the positive control paclitaxel, indicating our derivative may have the same biological targets, such as tubulin, as paclitaxel [32], while their binding modes and regulating functions might be different. It was documented in previous studies that paclitaxel bound to the microtubule at the taxoid site on the luminal face of β-tubulin and maintained the stability and destroyed the dynamics of the microtubule, thus leading to mitotic catastrophe [33]. However, according to the “similarity principle” [34], our 2,5-DKPs might have the same mechanism as plinabulin or its analogs, which acted as a tubulin binder near or at the colchicine binding site located at the interfacial region of the α- and β-tubulin studied using photoaffinity labeling [35,36] or computational modeling [37,38,39,40], leading to the inhibition of tubulin polymerization and the prevention of microtubule formation, thus resulting in the apoptosis of cancer cells. The detailed mechanism will be investigated in future studies.

## 3. Materials and Methods

### 3.1. Chemicals

Solvents (Guangzhou Chem. Re. Fac., Guangzhou, China) and chemicals (Macklin Com. Ltd., Shanghai, China) were commercially available in analytical grade and used without further purification. Analytical thin layer chromatography (TLC) analyses were performed on HSGF254 plates (Yantai Jiangyou Chem. Ltd., Yantai, China) which were visualized using UV light (254 nm) or I_2_ staining. Flash column chromatography was performed using silica gel (200–300 mesh, Yantai Jiangyou Chem. Ltd., Yantai, China). Melting points (mp) were determined using an SGW-X4 melting point instrument (Shanghai Jingke Ins. Co. Ltd., Shanghai, China) without correction. Mass spectrometry data were collected with a Bruker maXis/QTOF instrument (Bruker Corp., Karlsruhe, German) at a high resolution using ESI ionization. The NMR spectra were recorded on Bruker AC 500 or 700 NMR spectrometers (Bruker Corp., Karlsruhe, German) with TMS as an internal standard. The residual solvent peaks were used for the chemical shifts as an internal reference (ppm): ^1^H (CDCl_3_: δ 7.26); ^13^C (CDCl_3_: δ 77.0). HPLC was conducted on a Primaide instrument (1110 pump, HITACHI, Tokyo, Japan) and a Primaide 1430 diode array director (at 254 nm, HITACHI, Tokyo, Japan) with a C18 column, 5 μm, 4.6 × 250 mm (NanoChrom Technologies Co., Ltd., Suzhou, China) for the analysis of the purity of the target compounds (eluent: MeOH/H_2_O = 87:13).

#### 3.1.1. Synthesis of Compounds

##### General Procedure for the Synthesis of Products 1–16

1,4-diacetyl-2,5-diketopiperazine (50 mg, 0.25 mmol, 1.0 equiv.) and the first corresponding aldehydes (0.25 mmol, 1.0 equiv.) dissolved in 2 mL dry DMF were added into a 4 mL vial, followed by allyl bromide (54 μL, 0.63 mmol, 2.5 equiv.) and Cs_2_CO_3_ (205 mg, 0.63 mmol, 2.5 equiv.). The reaction was stirred at room temperature until the completion of the 1st aldol condensation and the alkylation of the allyl bromide. Then, the second corresponding aldehydes (0.5 mmol, 2.0 equiv.) were added, and the mixture was heated at 95 °C for about 4 h. The reaction mixture was added to water (15 mL) and extracted with AcOEt (5 mL × 3). The organic layer was dried over Na_2_SO_4_, filtered, and removed. The residues were purified by flash column chromatography on silica to afford the target products **1**–**5** and **8**–**16**. The procedure for the synthesis of compounds **6** and **7** was similar to the above synthetic method; however, the aldehydes (3.0 equiv.) were added just once in the first step. Compounds **3** [41] and **7** [28] are known, and their analytic data are identical to the reported values.

##### 1-allyl-6-((*Z*)-2-methoxybenzylidene)-3-((*Z*)-3-nitrobenzylidene)piperazine-2,5-dione (**1**)

Following the general procedure, product **1** was obtained in 24% yield as a slightly yellow solid. R_f_ = 0.15 (PE/AcOEt = 4:1), mp = 138–141 °C. ^1^H NMR (500 MHz, CDCl_3_) δ 9.65 (d, *J* = 28.5 Hz, 1H), 8.44 (d, *J* = 1.5 Hz, 1H), 8.04 (d, *J* = 8.2 Hz, 1H), 7.78 (d, *J* = 7.7 Hz, 1H), 7.58 (t, *J* = 8.0 Hz, 1H), 7.45–7.32 (m, 1H), 7.19 (d, *J* = 7.4 Hz, 1H), 7.07 (s, 1H), 7.00 (d, *J* = 7.5 Hz, 1H), 6.98–6.95 (m, 1H), 6.93 (d, *J* = 8.3 Hz, 1H), 5.52 (ddt, *J* = 16.3, 10.4, 6.0 Hz, 1H), 4.99 (d, *J* = 10.2 Hz, 1H), 4.74 (d, *J* = 17.1 Hz, 1H), 4.21 (d, *J* = 5.9 Hz, 2H), 3.86 (s, 3H).^13^C NMR (125 MHz, CDCl_3_) δ 161.4, 158.8, 157.2, 148.4, 135.0, 134.8, 131.2, 130.7, 130.3, 129.8, 128.1, 127.8, 123.7, 122.7, 122.3, 120.2, 118.8, 118.1, 114.9, 110.6, 55.4, 47.3 ppm. HRMS (ESI): *m/z* calcd. for C_22_H_20_N_3_O_5_ [M + H]^+^ 406.1397, found 406.1390; for C_21_H_19_N_3_O_5_Na [M + Na]^+^ 428.1217, found 428.1211. Purity: 95.2%.

##### 4-((*Z*)-(4-allyl-5-((*Z*)-2-methoxybenzylidene)-3,6-dioxopiperazin-2-ylidene)methyl)benzonitrile (**2**)

Following the general procedure, product **2** was obtained in 46% yield as a slightly yellow solid. R_f_ = 0.27 (PE/AcOEt = 4:1), mp = 175–178 °C. ^1^H NMR (500 MHz, CDCl_3_) δ 8.34 (s, 1H), 7.73 (d, *J* = 8.3 Hz, 2H), 7.56 (d, *J* = 8.2 Hz, 2H), 7.39–7.35 (m, 1H), 7.33 (s, 1H), 7.21 (d, *J* = 6.8 Hz, 1H), 7.01 (d, *J* = 7.9 Hz, 1H), 6.99 (t, *J* = 7.4 Hz, 1H), 6.94 (d, *J* = 8.3 Hz, 1H), 5.54 (ddt, *J* = 16.3, 10.2, 6.0 Hz, 1H), 5.02 (dd, *J* = 10.2, 1.1 Hz, 1H), 4.77 (dd, *J* = 17.1, 1.2 Hz, 1H), 4.25 (d, *J* = 6.0 Hz, 2H), 3.87 (s, 3H). ^13^C NMR (126 MHz, CDCl_3_) δ 160.3, 158.6, 157.4, 137.8, 132.9, 131.2, 130.9, 130.4, 129.3, 128.1, 127.8, 122.5, 120.3, 119.4 118.4, 118.3, 114.6, 112.0, 110.7, 55.5, 47.5 ppm. HRMS (ESI): *m/z* calcd. for C_23_H_20_N_3_O_3_ [M + H]^+^ 386.1499, found 386.1498. Purity: 89.0%.

##### 1-allyl-6-((*Z*)-3-bromobenzylidene)-3-((*Z*)-3-nitrobenzylidene)piperazine-2,5-dione (**4**)

Following the general procedure, product **4** was obtained in 63% yield as a slightly yellow solid. R_f_ = 0.18 (PE/AcOEt = 4:1), mp = 182–185 °C. ^1^H NMR (500 MHz, CDCl_3_) δ 9.61 (s, 1H), 8.48 (s, 1H), 8.09 (d, *J* = 8.1 Hz, 1H), 7.75 (d, *J* = 7.7 Hz, 1H), 7.61 (t, *J* = 8.0 Hz, 1H), 7.51 (d, *J* = 8.0 Hz, 1H), 7.41 (s, 1H), 7.30 (t, *J* = 7.8 Hz, 1H), 7.21 (d, *J* = 7.7 Hz, 1H), 7.10 (s, 1H), 6.81 (s, 1H), 5.54–5.50 (m, 1H), 5.05 (d, *J* = 10.2 Hz, 1H), 4.78 (d, *J* = 17.1 Hz, 1H), 4.23 (d, *J* = 5.8 Hz, 2H). ^13^C NMR (125 MHz, CDCl_3_) δ 160.7, 158.9, 148.6, 135.7, 135.1, 134.7, 132.0, 131.0, 130.1, 130.0, 128.8, 127.8, 127.8, 123.5, 123.0, 122.6, 120.5, 118.5, 115.6, 48.1 ppm. HRMS (ESI): *m/z* calcd. for C_21_H_17_BrN_3_O_4_ [M + H]^+^ 454.0397, found 454.0400; purity: 94.6%.

##### 1-allyl-3-((*Z*)-2,3-dichlorobenzylidene)-6-((*Z*)-2-(trifluoromethyl)benzylidene)piperazine-2,5-dione (**5**)

Following the general procedure, product **5** was obtained in 21% yield as an orange solid. R_f_ = 0.64 (PE/AcOEt = 4:1), mp = 109–111 °C. ^1^H NMR (700 MHz, CDCl_3_) δ 8.94 (s, 1H), 7.74 (d, *J* = 7.9 Hz, 1H), 7.57 (t, *J* = 7.6 Hz, 1H), 7.48 (t, *J* = 7.7 Hz, 1H), 7.41–7.38 (m, 2H), 7.35 (d, *J* = 7.6 Hz, 1H), 7.26 (t, *J* = 7.9 Hz, 1H), 7.24 (s, 1H), 7.13 (s, 1H), 5.56–5.46 (m, 1H), 5.00 (dd, *J* = 10.3, 1.1 Hz, 1H), 4.76 (dd, *J* = 17.1, 1.1 Hz, 1H), 4.09 (d, *J* = 7.0 Hz, 2H).^13^C NMR (176 MHz, CDCl_3_) δ 159.2, 158.4, 134.1, 133.3, 132.6, 132.5, 131.6, 130.8, 130.4 (d, *J*_C–F_ = 2.9 Hz), 129.8, 128.8, 128.6 (q, *J*_C–F_ = 30.3 Hz), 127.8, 127.6, 127.3, 126.3 (d, *J*_C–F_ = 4.6 Hz), 123.7 (q, *J*_C–F_ = 273.9 Hz), 117.9, 117.7, 114.8, 48.1 ppm. HRMS (ESI): *m/z* calcd. for C_22_H_16_Cl_2_F_3_N_2_O_2_ [M + H]^+^ 467.0538, found 467.0535. Purity: 97.2%.

##### 1-allyl-3,6-bis((*Z*)-3-(trifluoromethyl)benzylidene)piperazine-2,5-dione (**6**)

Following the general procedure, product **6** was obtained in 39% yield as a white solid. R_f_ = 0.64 (PE/AcOEt = 4:1), mp = 154–155 °C. ^1^H NMR (500 MHz, CDCl_3_) δ 8.97 (s, 1H), 7.71 (s, 1H), 7.63 (s, 2H), 7.60–7.51 (m, 4H), 7.47 (d, *J* = 7.2 Hz, 1H), 7.10 (d, *J* = 7.0 Hz, 2H), 5.53–5.48 (m, 1H), 5.03 (d, *J* = 10.2 Hz, 1H), 4.72 (d, *J* = 17.0 Hz, 1H), 4.23 (s, 2H). ^13^C NMR (126 MHz, CDCl_3_) δ 160.0, 159.0, 134.7, 133.7, 132.3, 131.9, 131.7(q, *J*_C–F_ = 37.2 Hz), 131.1(q, *J*_C–F_ = 32.7 Hz), 131.0, 129.8, 129.3, 129.0, 126.9, 126.0 (d, *J*_C–F_ = 3.6 Hz), 125.6 (d, *J* = 3.7 Hz), 125.4 (d, *J* = 3.5 Hz), 125.3 (d, *J* = 3.5 Hz), 123.7 (q, *J*_C–F_ = 272.7 Hz), 123.7 (q, *J*_C–F_ = 272.6 Hz). 120.3, 118.4, 116.8, 48.0 ppm. HRMS (ESI): *m/z* calcd. for C_23_H_17_F_6_N_2_O_2_ [M + H]^+^ 467.1189, found 467.1199. Purity: 90.6%.

##### (*Z*)-1-allyl-3-(furan-2-ylmethylene)-6-((*Z*)-2-methoxybenzylidene)piperazine-2,5-dione (**8**)

Following the general procedure, product **8** was obtained in 65% yield as a slightly yellow oil. R_f_ = 0.31 (PE/AcOEt = 4:1), ^1^H NMR (500 MHz, CDCl_3_) δ 9.14 (s, 1H), 7.57 (s, 1H), 7.34 (s, 1H), 7.32 (d, *J* = 8.4 Hz, 1H), 7.18 (d, *J* = 7.3 Hz, 1H), 6.95 (t, *J* = 7.4 Hz, 1H), 6.90 (d, *J* = 8.3 Hz, 1H), 6.78 (s, 1H), 6.57 (d, *J* = 3.4 Hz, 1H), 6.51 (dd, *J* = 3.4, 1.8 Hz, 1H), 5.55–5.50 (m, 1H), 4.98 (d, *J* = 11.2 Hz, 1H), 4.74 (d, *J* = 17.1 Hz, 1H), 4.23 (d, *J* = 5.9 Hz, 2H), 3.83 (s, 3H). ^13^C NMR (125 MHz, CDCl_3_) δ 159.5, 158.9, 157.3, 150.8, 143.9, 131.4, 130.4, 130.3, 128.3, 123.7, 123.0, 120.1, 118.1, 117.9, 114.2, 112.3, 110.5, 103.4, 55.4, 47.4 ppm. HRMS (ESI): *m/z* calcd. for C_20_H_19_N_2_O_4_ [M + H]^+^ 351.1339, found 351.1346. Purity: 93.1%.

##### (*Z*)-1-allyl-6-((*Z*)-2-methoxybenzylidene)-3-(thiophen-2-ylmethylene)piperazine-2,5-dione (**9**)

Following the general procedure, product **9** was obtained in 46% yield as a slightly yellow oil. R_f_ = 0.28 (PE/AcOEt = 4:1), ^1^H NMR (500 MHz, CDCl_3_) δ 8.06 (s, 1H), 7.45 (d, *J* = 5.1 Hz, 1H), 7.35–7.32 (m, 2H), 7.28 (d, *J* = 3.6 Hz, 1H), 7.20 (d, *J* = 5.0 Hz, 2H), 7.13 (dd, *J* = 5.1, 3.7 Hz, 1H), 6.96 (t, *J* = 7.5 Hz, 1H), 6.91 (d, *J* = 8.2 Hz, 1H), 5.56–5.51 (m, 1H), 4.99 (d, *J* = 11.3 Hz, 1H), 4.75 (d, *J* = 17.1 Hz, 1H), 4.22 (d, *J* = 6.0 Hz, 2H), 3.84 (s, 3H). ^13^C NMR (125 MHz, CDCl_3_) δ 159.9, 159.1, 157.3, 135.8, 131.4, 130.5, 130.3, 129.7, 128.3, 128.1, 127.6, 124.1, 122.7, 120.2, 118.4, 118.0, 110.6, 110.4, 55.4, 47.3 ppm. HRMS (ESI): *m/z* calcd. For C_20_H_19_N_2_O_3_S [M +H]^+^ 367.1111, found 367.1114. Purity: 98.5%.

##### (*Z*)-1-allyl-6-((*Z*)-2-methoxybenzylidene)-3-(12yridine-2-ylmethylene)piperazine-2,5-dione (**10**)

Following the general procedure, product **10** was obtained in 38% yield as a slightly yellow oil. R_f_ = 0.22 (PE/AcOEt = 4:1), ^1^H NMR (500 MHz, CDCl_3_) δ 12.66 (s, 1H), 8.63 (s, 1H), 7.71 (t, *J* = 7.7 Hz, 1H), 7.38 (s, 1H), 7.33 (t, *J* = 7.9 Hz, 2H), 7.19 (d, *J* = 7.3 Hz, 2H), 6.96 (t, *J* = 7.4 Hz, 1H), 6.90 (d, *J* = 8.3 Hz, 1H), 6.79 (s, 1H), 5.58–5.50 (m, 1H), 4.99 (d, *J* = 10.2 Hz, 1H), 4.75 (d, *J* = 17.1 Hz, 1H), 4.26 (d, *J* = 5.6 Hz, 2H), 3.84 (s, 3H). ^13^C NMR (125 MHz, CDCl_3_) δ 159.5, 158.8, 157.2, 154.9, 148.4, 136.9, 131.4, 130.9, 130.3, 130.3, 128.6, 125.9, 123.2, 122.0, 120.1, 118.2, 117.9, 110.6, 109.8, 55.4, 47.4 ppm. HRMS (ESI): *m/z* calcd. for C_21_H_20_N_3_O_3_ [M + H]^+^ 362.1499, found 362.1507. Purity: 90.7%.

##### (*Z*)-1-allyl-6-((*Z*)-2-methoxybenzylidene)-3-(naphthalen-1-ylmethylene)piperazine-2,5-dione (**11**)

Following the general procedure, product **11** was obtained in 58% yield as a slightly yellow solid. R_f_ = 0.34 (PE/AcOEt = 4:1), mp = 80–82 °C. ^1^H NMR (700 MHz, CDCl_3_) δ 8.13 (s, 1H), 8.01–8.00 (m, 1H), 7.88–7.86 (m, 1H), 7.85 (d, *J* = 8.1 Hz, 1H), 7.56–7.50 (m, 5H), 7.37–7.35 (m, 1H), 7.30 (s, 1H), 7.25 (d, *J* = 7.2 Hz, 1H), 7.00 (t, *J* = 7.5 Hz, 1H), 6.93 (d, *J* = 8.3 Hz, 1H), 5.59 (ddt, *J* = 16.3, 10.3, 6.0 Hz, 1H), 5.03 (dd, *J* = 10.2, 1.0 Hz, 1H), 4.80 (dd, *J* = 17.1, 1.2 Hz, 1H), 4.31 (d, *J* = 6.0 Hz, 2H), 3.87 (s, 3H). ^13^C NMR (125 MHz, CDCl_3_) δ 159.8, 158.9, 157.3, 133.8, 131.5, 131.4, 130.5, 130.4, 129.8, 129.3, 128.7, 128.2, 127.6, 126.9, 126.6, 126.0, 125.5, 124.5, 122.9, 120.2, 118.5, 118.1, 115.3, 110.6, 55.4, 47.4 ppm. HRMS (ESI): *m/z* calcd. For C_26_H_23_N_2_O_3_ [M + H]^+^ 411.1703, found 411.1696. Purity: 98.4%.

##### (*Z*)-1-allyl-6-((*Z*)-2-methoxybenzylidene)-3-(quinoline-2-ylmethylene)piperazine-2,5-dione (**12**)

Following the general procedure, product **12** was obtained in 56% yield as a slightly yellow solid. R_f_ = 0.33 (PE/AcOEt = 4:1), mp = 170–173 °C. ^1^H NMR (500 MHz, CDCl_3_) δ 13.23 (s, 1H), 8.18–8.14 (m, 2H), 7.78 (d, *J* = 8.1 Hz, 1H), 7.74 (t, *J* = 7.1 Hz, 1H), 7.54 (t, *J* = 7.5 Hz, 1H), 7.43 (t, *J* = 5.1 Hz, 2H), 7.34 (t, *J* = 7.8 Hz, 1H), 7.22 (d, *J* = 7.3 Hz, 1H), 6.97 (t, *J* = 7.4 Hz, 1H), 6.92 (d, *J* = 10.4 Hz, 2H), 5.61–5.53 (m, 1H), 5.01 (d, *J* = 11.2 Hz, 1H), 4.79 (d, *J* = 18.2 Hz, 1H), 4.30 (d, *J* = 5.9 Hz, 2H), 3.86 (s, 3H). ^13^C NMR (125 MHz, CDCl_3_) δ 159.6, 158.6, 157.3, 155.1, 146.8, 136.8, 132.2, 131.4, 130.4, 130.4, 130.2, 128.8, 128.6, 127.5, 127.0, 126.7, 123.8, 123.2, 120.2, 118.4, 117.9, 110.6, 109.3, 55.4, 47.5 ppm. HRMS (ESI): *m/z* calcd. for C_25_H_22_N_3_O_3_ [M + H]^+^ 412.1656, found 412.1660. Purity: 91.1%.

##### (*Z*)-1-allyl-6-((*Z*)-2-methoxybenzylidene)-3-((6-methoxynaphthalen-2-yl)methylene)piperazine-2,5-dione (**13**)

Following the general procedure, product **13** was obtained in 45% yield as a slightly yellow solid. R_f_ = 0.33 (PE/AcOEt = 4:1), mp = 156–158 °C. ^1^H NMR (500 MHz, CDCl_3_) δ 8.24 (s, 1H), 7.86 (s, 1H), 7.77 (dd, *J* = 15.0, 8.7 Hz, 2H), 7.48 (dd, *J* = 8.5, 1.5 Hz, 1H), 7.40–7.33 (m, 1H), 7.32 (s, 1H), 7.23 (d, *J* = 7.0 Hz, 1H), 7.21–7.16 (m, 2H), 7.12 (d, *J* = 2.3 Hz, 1H), 6.98 (t, *J* = 7.4 Hz, 1H), 6.92 (d, *J* = 8.3 Hz, 1H), 5.57 (ddt, *J* = 16.3, 10.3, 6.0 Hz, 1H), 5.01 (dd, *J* = 10.2, 1.1 Hz, 1H), 4.78 (dd, *J* = 17.1, 1.2 Hz, 1H), 4.26 (d, *J* = 6.0 Hz, 2H), 3.93 (s, 3H), 3.86 (s, 3H). ^13^C NMR (126 MHz, CDCl_3_) δ 160.2, 159.4, 158.6, 157.4, 134.4, 131.5, 130.5, 130.4, 129.7, 128.8, 128.5, 128.2, 127.9, 127.9, 126.5, 125.6, 122.9, 120.2, 119.8, 118.3, 118.0, 117.7, 110.6, 105.8, 55.5, 55.4, 47.4 ppm. HRMS (ESI): *m/z* calcd. for C_27_H_25_N_2_O_4_ [M + H]^+^ 441.1809, found 441.1815. Purity: 95.1%.

##### (*Z*)-1-allyl-3-(naphthalen-1-ylmethylene)-6-((*Z*)-2-(trifluoromethyl)benzylidene)piperazine-2,5-dione (**14**)

Following the general procedure, product **14** was obtained in 30% yield as a slightly yellow solid. R_f_ = 0.71 (PE/AcOEt = 4:1), mp = 98–101 °C. ^1^H NMR (700 MHz, CDCl_3_) δ 8.52 (s, 1H), 8.04–7.94 (m, 1H), 7.83 (t, *J* = 7.0 Hz, 2H), 7.74 (d, *J* = 7.9 Hz, 1H), 7.59 (s, 1H), 7.58–7.55 (m, 2H), 7.55–7.50 (m, 3H), 7.48 (t, *J* = 7.7 Hz, 1H), 7.38 (d, *J* = 7.6 Hz, 1H), 7.33 (s, 1H), 5.57 (ddd, *J* = 22.7, 10.6, 5.6 Hz, 1H), 5.04 (dd, *J* = 10.3, 0.9 Hz, 1H), 4.81 (dd, *J* = 17.1, 1.0 Hz, 1H), 4.15 (s, 2H).^13^C NMR (176 MHz, CDCl_3_) δ 158.9, 158.8, 133.8, 133.0, 131.6, 131.5, 131.1, 130.6, 130.1, 129.6, 129.4, 128.7 (q, *J*_C–F_ = 30.6 Hz), 128.7, 127.0, 126.9, 126.6, 126.4 (d, *J*_C–F_= 3.1 Hz), 125.5, 124.5, 123.8 (q, *J*_C–F_ = 274.3 Hz), 117.8, 117.3, 116.4, 48.1 ppm. HRMS (ESI): *m/z* calcd. for C_26_H_20_F_3_N_2_O_2_ [M + H]^+^ 449.1471, found 449.1480. Purity: 93.9%.

##### (*Z*)-1-allyl-3-(naphthalen-1-ylmethylene)-6-((*Z*)-3-(trifluoromethyl)benzylidene)piperazine-2,5-dione (**15**)

Following the general procedure, product **15** was obtained in 37% yield as a slightly yellow solid. R_f_ = 0.67 (PE/AcOEt = 4:1), mp = 111–113 °C. ^1^H NMR (700 MHz, CDCl_3_) δ 4 (d, *J* = 19.6 Hz, 1H), 8.02–7.97 (m, 1H), 7.87 (dd, *J* = 11.7, 5.6 Hz, 2H), 7.62 8.3 (t, *J* = 7.6 Hz, 1H), 7.60 (d, *J* = 9.1 Hz, 1H), 7.56 (dd, *J* = 6.6, 1.3 Hz, 2H), 7.55 (dd, *J* = 5.3, 2.3 Hz, 2H), 7.54 (s, 1H), 7.52 (t, *J* = 6.3 Hz, 1H), 7.50 (t, *J* = 6.0 Hz, 1H), 7.15 (s, 1H), 5.54 (ddt, *J* = 16.1, 10.3, 5.8 Hz, 1H), 5.05 (dd, *J* = 10.2, 1.1 Hz, 1H), 4.75 (dd, *J* = 17.1, 1.1 Hz, 1H), 4.28 (d, *J* = 5.8 Hz, 2H).^13^C NMR (176 MHz, CDCl_3_) δ 159.2, 159.0, 135.0, 133.8, 132.3, 131.5, 131.1, 130.9 (q, *J*_C–F_ = 32.8 Hz), 129.6, 129.4, 129.3, 128.9, 128.6, 127.1, 126.9, 126.6, 126.2, 126.0 (d, *J*_C–F_ = 2.4 Hz), 125.4, 125.2 (d, *J*_C–F_ = 2.4 Hz), 124.5, 122.1 (d, *J*_C–F_ = 272.6 Hz), 119.6, 118.2, 116.6, 48.0 ppm. HRMS (ESI): *m/z* calcd. for C_26_H_20_F_3_N_2_O_2_ [M + H]^+^ 449.1471, found 449.1475. Purity: 87.8%.

##### (*Z*)-1-allyl-6-((*Z*)-2-fluorobenzylidene)-3-((6-methoxynaphthalen-2-yl)methylene)piperazine-2,5-dione (**16**)

Following the general procedure, product **16** was obtained in 67% yield as a bright yellow solid. R_f_ = 0.42 (PE/AcOEt = 4:1), mp = 152–155 °C. ^1^H NMR (500 MHz, CDCl_3_) δ 8.71 (s, 1H), 7.87 (s, 1H), 7.77 (d, *J* = 8.5 Hz, 1H), 7.74 (d, *J* = 9.0 Hz, 1H), 7.48 (dd, *J* = 8.5, 1.5 Hz, 1H), 7.36 (td, *J* = 7.3, 1.5 Hz, 1H), 7.26–7.22 (m, 1H), 7.21 (s, 1H), 7.19–7.16 (m, 1H), 7.16–7.13 (m, 1H), 7.11 (t, *J* = 9.2 Hz, 1H), 7.07 (d, *J* = 9.3 Hz, 2H), 5.53 (ddt, *J* = 16.2, 10.3, 5.9 Hz, 1H), 5.01 (dd, *J* = 10.2, 1.0 Hz, 1H), 4.74 (dd, *J* = 17.1, 1.1 Hz, 1H), 4.28 (d, *J* = 5.9 Hz, 2H), 3.88 (s, 3H). ^13^C NMR (126 MHz, CDCl_3_) δ 160.0 (d, *J*_C–F_ = 251.1 Hz), 159.7, 159.3, 158.6, 134.4, 131.2, 130.7, 130.7, 130.0, 129.7, 128.7, 128.0, 128.0, 127.8, 126.5, 125.2, 123.9 (d, *J*_C–F_ = 3.3 Hz), 122.2 (d, *J*_C–F_ = 14.7 Hz), 119.7, 118.6, 118.2, 115.7 (d, *J*_C–F_ = 21.3 Hz), 114.4, 105.7, 55.3, 47.2. HRMS (ESI): *m/z* calcd. for C_26_H_22_FN_2_O_3_ [M + H]^+^ 429.1609 found 429.1601. Purity: 95.2%.

#### 3.1.2. Liposolubility Tests

In 1.5 mL vials containing 1.0 mg of the synthetic compound, the solvent (DMSO, DMF, AcOEt, DCM, chloroform, or acetone) was then added dropwise, and the mixture was stirred simultaneously at room temperature until the complete dissolubility of the compound. The liposolubilites for all compounds were estimated and calculated on the basis of the volume (less than 1.0 mL each) of the above solvents used.

### 3.2. Biological Evaluations

#### 3.2.1. Cytotoxicity Bioassay

Cell viability was analyzed by a 3-(4,5-dimethyl-2-thiazolyl)-2,5-diphenyl tetrazolium bromide (MTT) assay as previously described [42]. In brief, cells were seeded in a 96-well plate at a density of 3 × 10^3^ per well treated with compounds (0.039, 0.078, 0.156, 0.312, 0.625, 1.25, 2.5, 5, 10 μM) in an incubator (5% CO_2_, 37 ℃) for 48 h. The OD_570_ values were measured using an ELISA Reader (M1000 Pro, TECAN, Männedorf, Switzerland). The experiment was independently repeated three times.

#### 3.2.2. Apoptosis and Cell Cycle Assay

A549, Hela cells and VK2/E6E7(from Southern Medical University, Guangzhou, China) were seeded and incubated in six-well plates at a density of 2.0 × 10^5^ cells per well and treated with DMSO (0.1 %, *v*/*v*), paclitaxel (1.0 μM) or compound **11** (0.25, 0.5, 1.0 μM) for 48 h. The cells were then collected and stained with both annexin V-FITC and PI solutions following the manufacturer’s protocol (BMS500FI-300, Thermo Fisher Scientific, Waltham, MA, USA). Apoptotic rates and cell cycle distribution of both cells were examined and analyzed by a flow cytometer (BD Fac SCanto II, San Jose, CA, USA). Three parallel experiments were performed for each concentration.

## 4. Conclusions

Sixteen 2,5-DKPs with different functions substituted at the 3,6 positions of the 2,5-DKP ring were designed, synthesized, and investigated as anticancer agents against cell lines A549 and Hela. 2-OMe on the phenyl group at the 6 position and the suitable size or skeleton substituents at the 3 position on the 2,5-DKP ring might have combined impacts on the anticancer activities of the derivatives. The one-pot method for the synthesis of these derivatives is simple and operationally convenient, which is also a guarantee of providing enough amounts of the active derivatives for further druggability assessments. The biological evaluation disclosed that the activities of most derivatives were not obviously improved except for compound **11**, which is a promising skeleton for further development to explore highly active anticancer agents; therefore, our ongoing studies are now mainly focusing on this compound.

## Figures and Tables

**Table 1:**
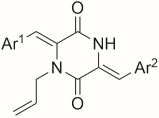
IC_50_ values of compounds against A549 and Hela cancer cell lines.

Compound	IC_50_/μM ^a^	
	A549	Hela
**4m**	2.3 ± 0.2	1.6 ± 0.2
**1**	>10	>10
**2**	>10	>10
**3**	>10	>10
**4**	>10	>10
**5**	>10	>10
**6**	>10	8.9 ± 0.7
**7**	>10	>10
**8**	7.3 ± 1.2	5.9 ± 1.6
**9**	3.7 ± 0.4	4.8 ± 1.4
**10**	5.6 ± 1.0	4.7 ± 2.8
**11**	1.2 ± 0.1	0.7 ± 0.2
**12**	>10	6.2 ± 0.4
**13**	>10	>10
**14**	>10	3.9 ± 0.2
**15**	>10	>10
**16**	>10	>10
Paclitaxel ^b^	0.004 ± 0.2	0.004 ± 0.8

^a^ All results are presented as mean ± standard deviation (SD) from three parallel experiments. ^b^ Paclitaxel as a positive control.

## Data Availability

The original data presented in the study are included in the article/Supplementary Materials; further inquiries can be directed to the corresponding author.

## Supplementary Materials

The following supporting information can be downloaded at: https://www.mdpi.com/article/10.3390/md21060325/s1, Figures S1–S28: The NMR spectra for all the derivatives; Figure S29: 1H-1H NOESY of 11; Figures S30–S45: HPLC spectra for compounds **1**–**16**. References [28,41] are cited in the Supplementary Materials.
