# Supplementary material for: Design, Synthesis, and Anticancer Activity of Novel 3,6-Diunsaturated 2,5-Diketopiperazines
Source: Mar Drugs. 2023 May 26;21(6):325. doi: 10.3390/md21060325 (PMC10301345; doi:10.3390/md21060325)
Supplement: Supplementary file 1 [file marinedrugs-21-00325-s001.zip › marinedrugs-2391444-supplementary.pdf]

Copies of NMR spectra for the derivatives

Note: **3** and **7** are known compounds[1-2]

Figure **S1.** ^{1}H NMR of **1.**

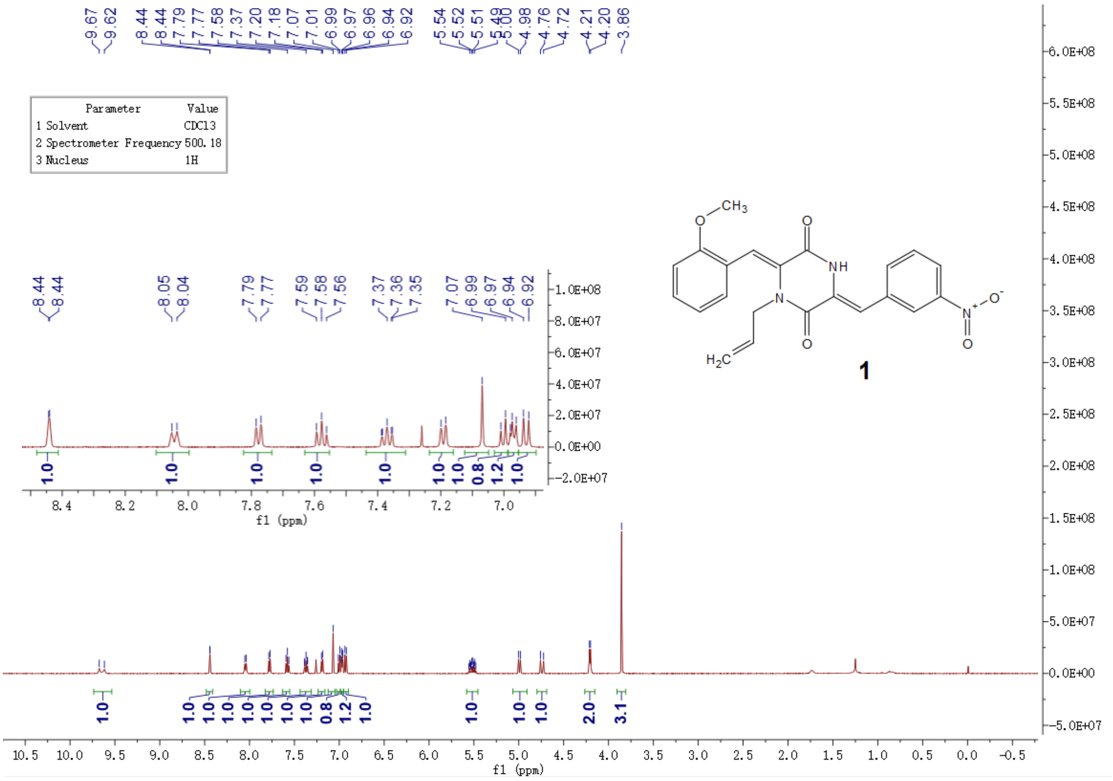

Figure **S2.** ^{13}C NMR of **1.**

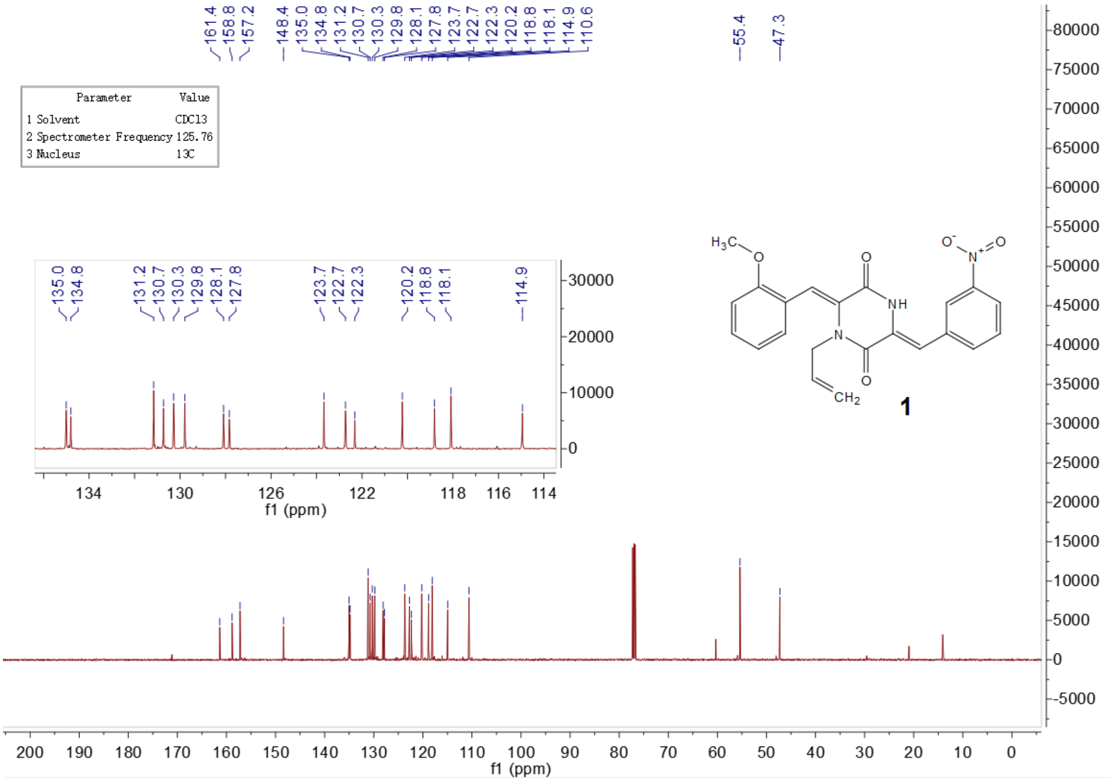

Figure **S3.** ^{1}H NMR of **2.**

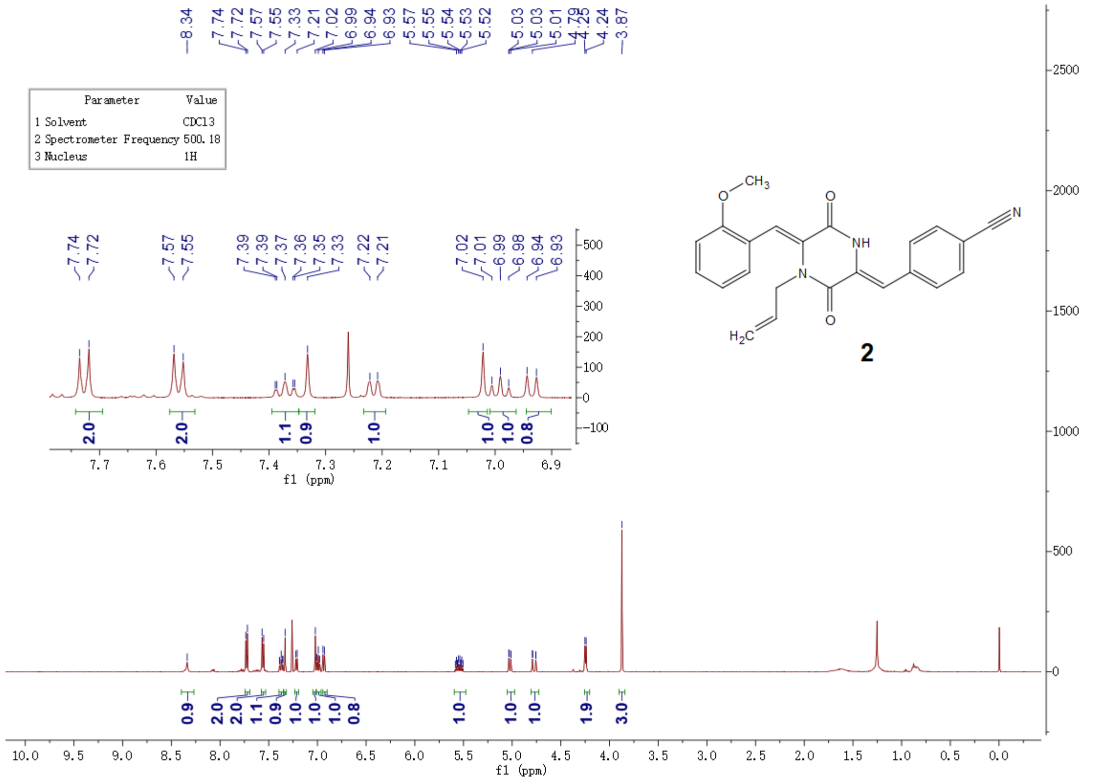

Figure **S4.** ^{13}C NMR of **2.**

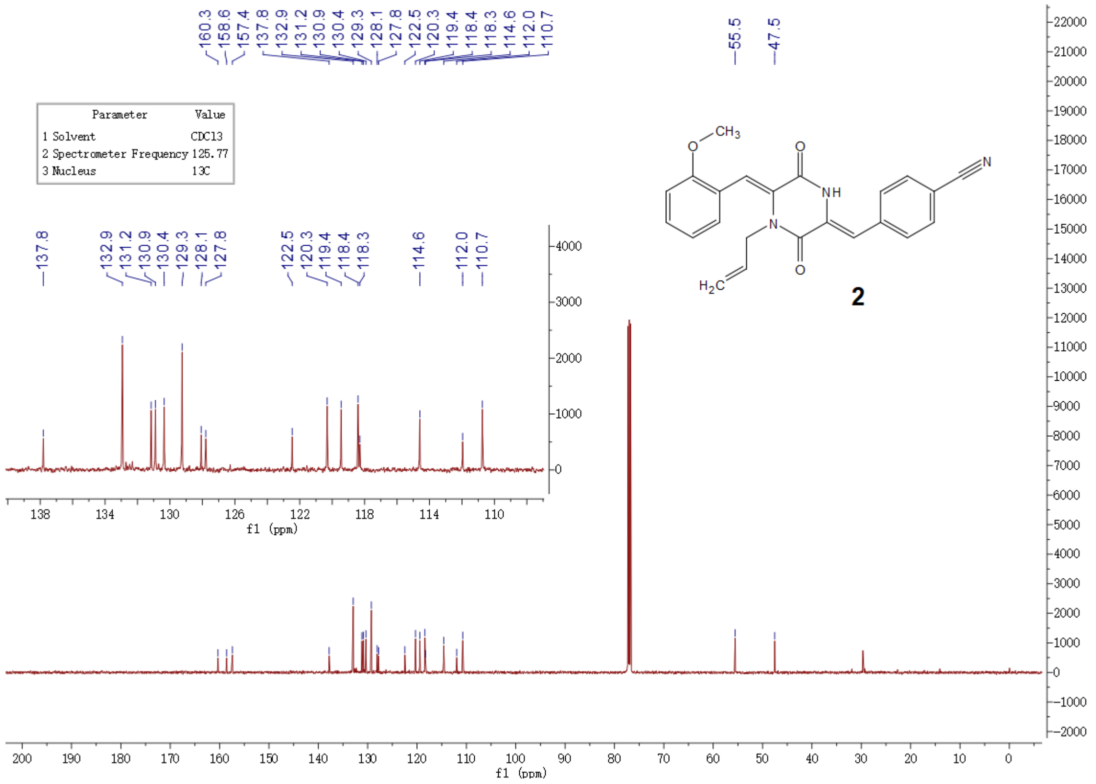

Figure **S5.** ^{1}H NMR of **4.**

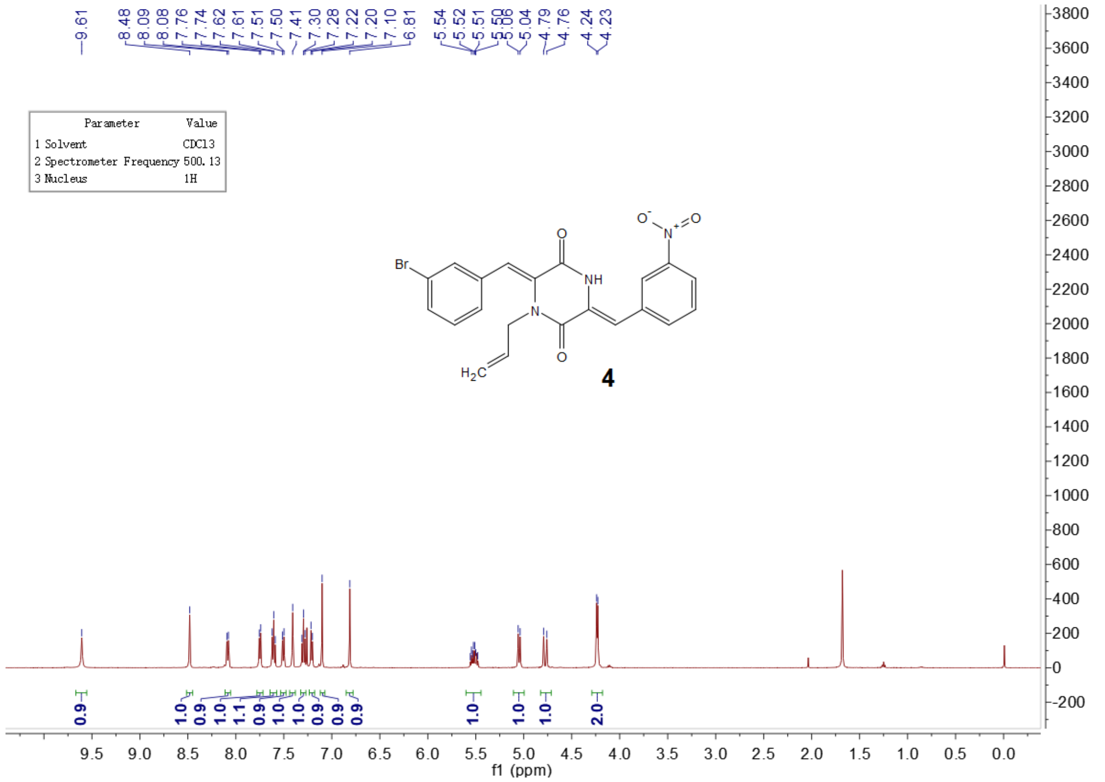

Figure **S6.** ^{13}C NMR of **4.**

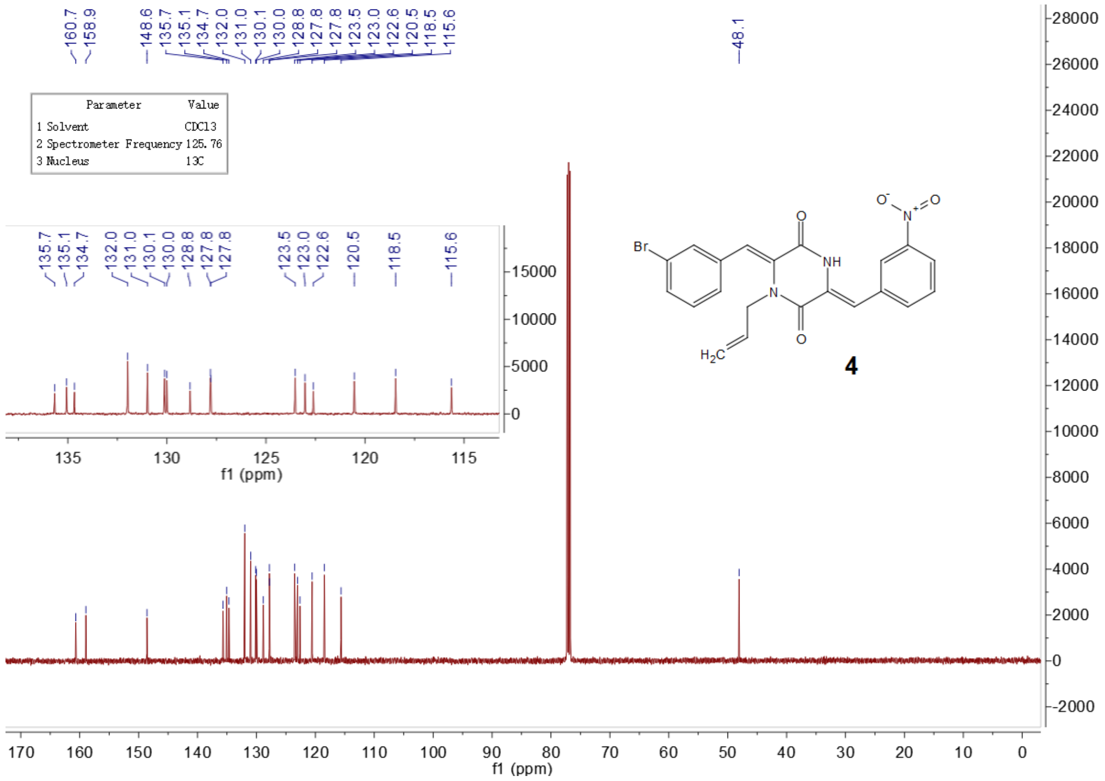

Figure **S7.** ^{1}H NMR of **5.**

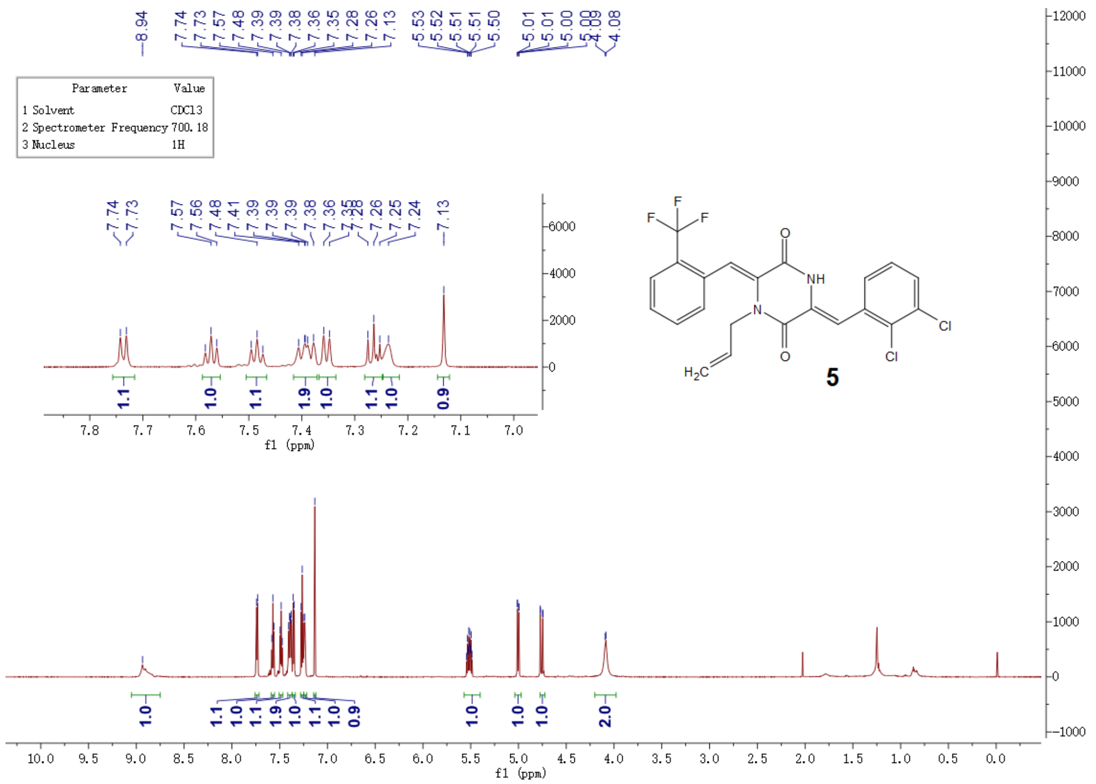

Figure **S8.** ^{13}C NMR of **5.**

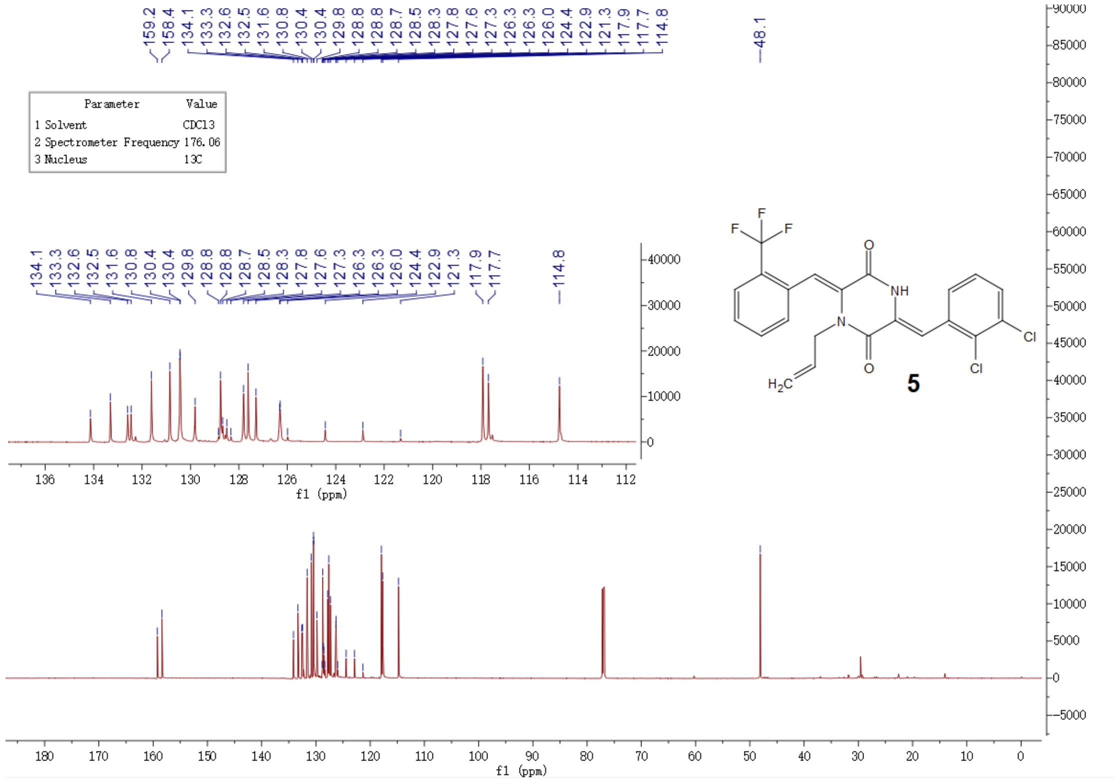

Figure **S9.** ^{1}H NMR of **6.**

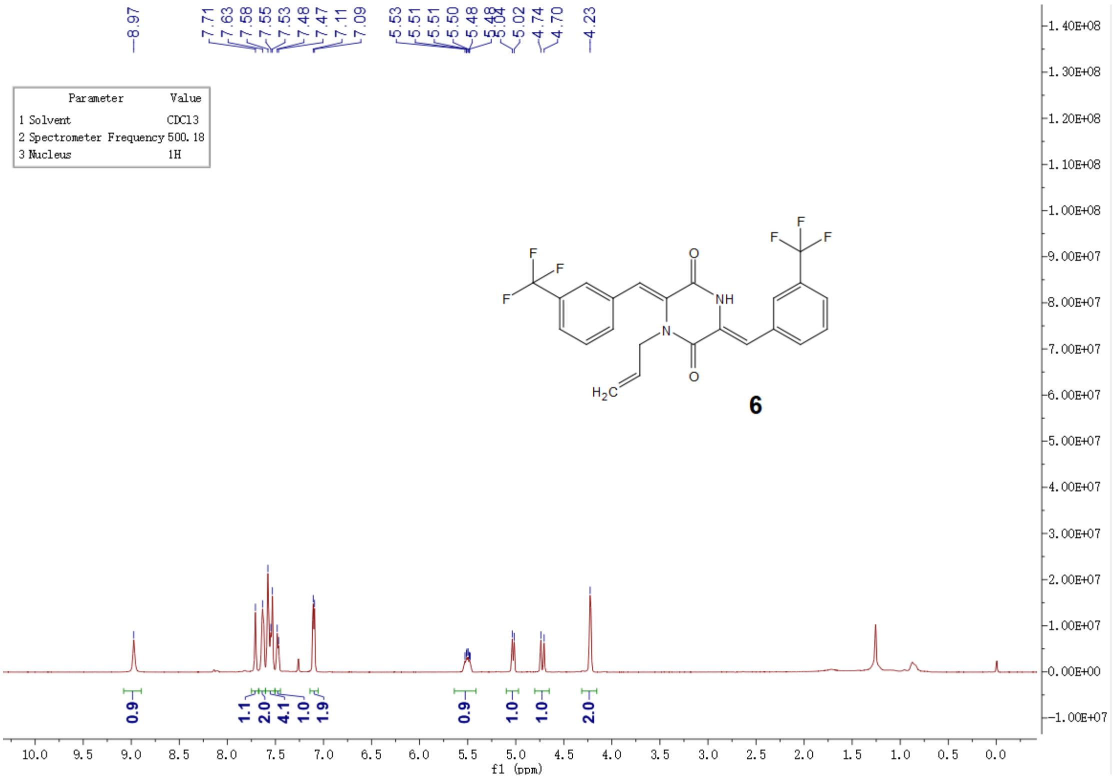

Figure **S10.** ^{13}C NMR of **6.**

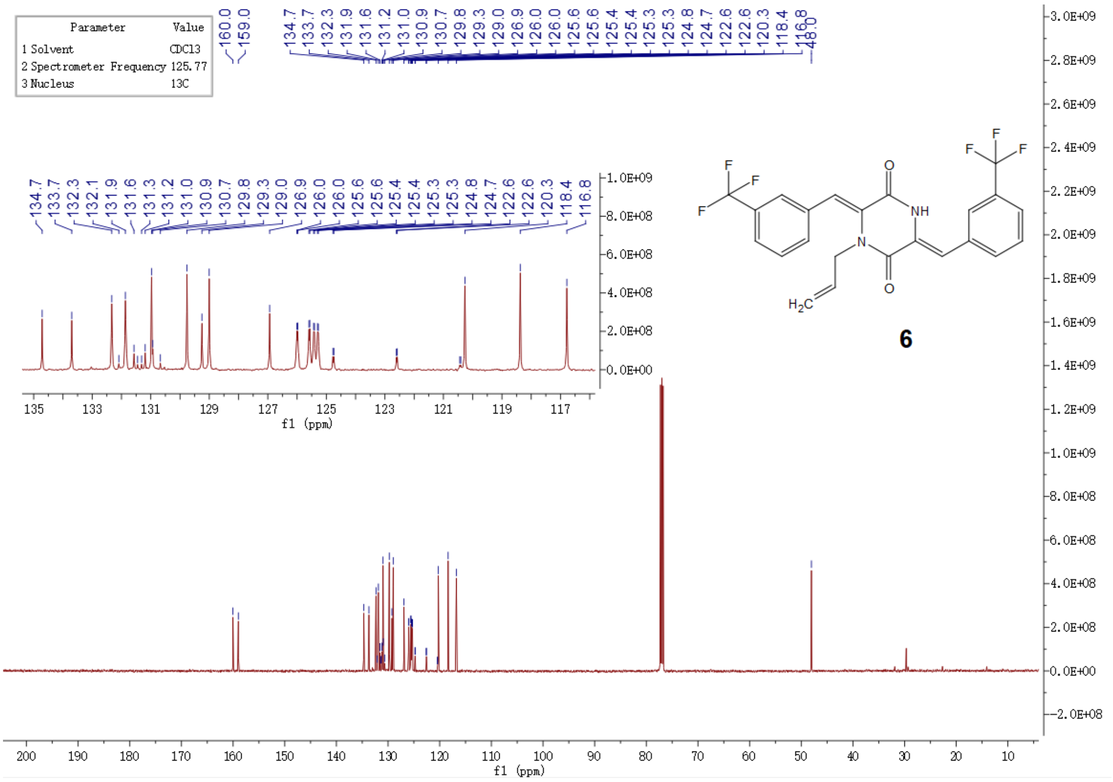

Figure **S11.** ^{1}H NMR of **8.**

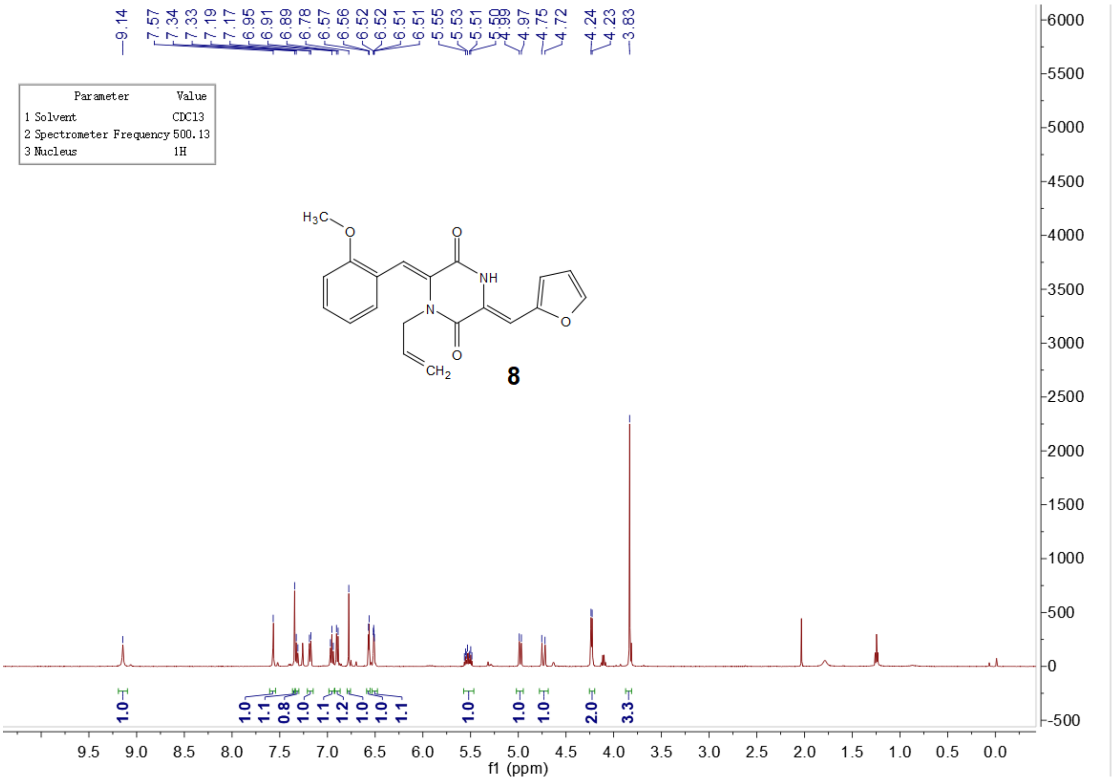

Figure **S12.** ^{13}C NMR of **8.**

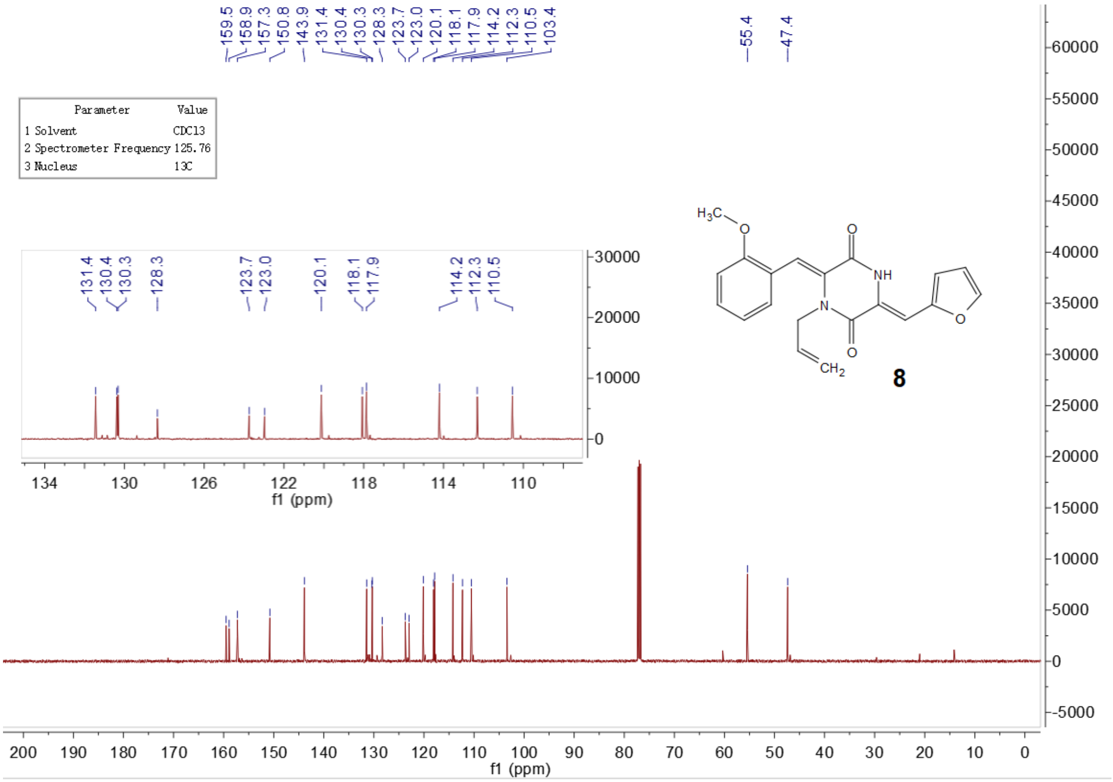

Figure **S13.** ^{1}H NMR of **9.**

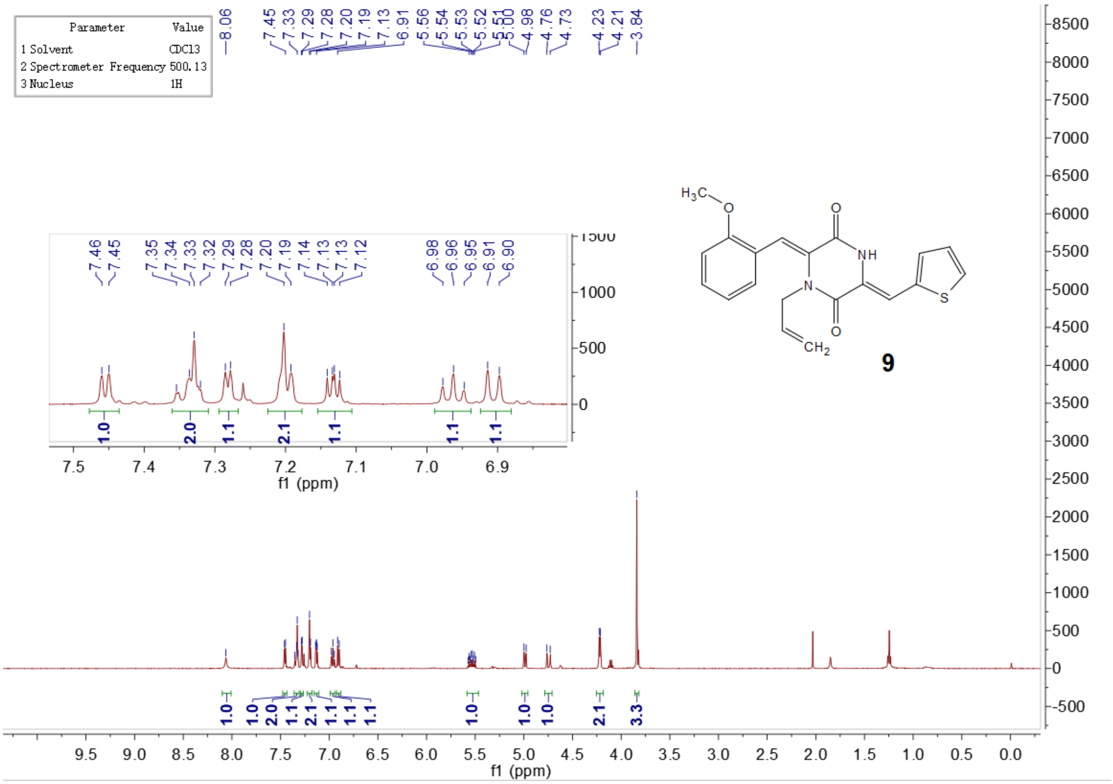

Figure **S14.** ^{13}C NMR of **9.**

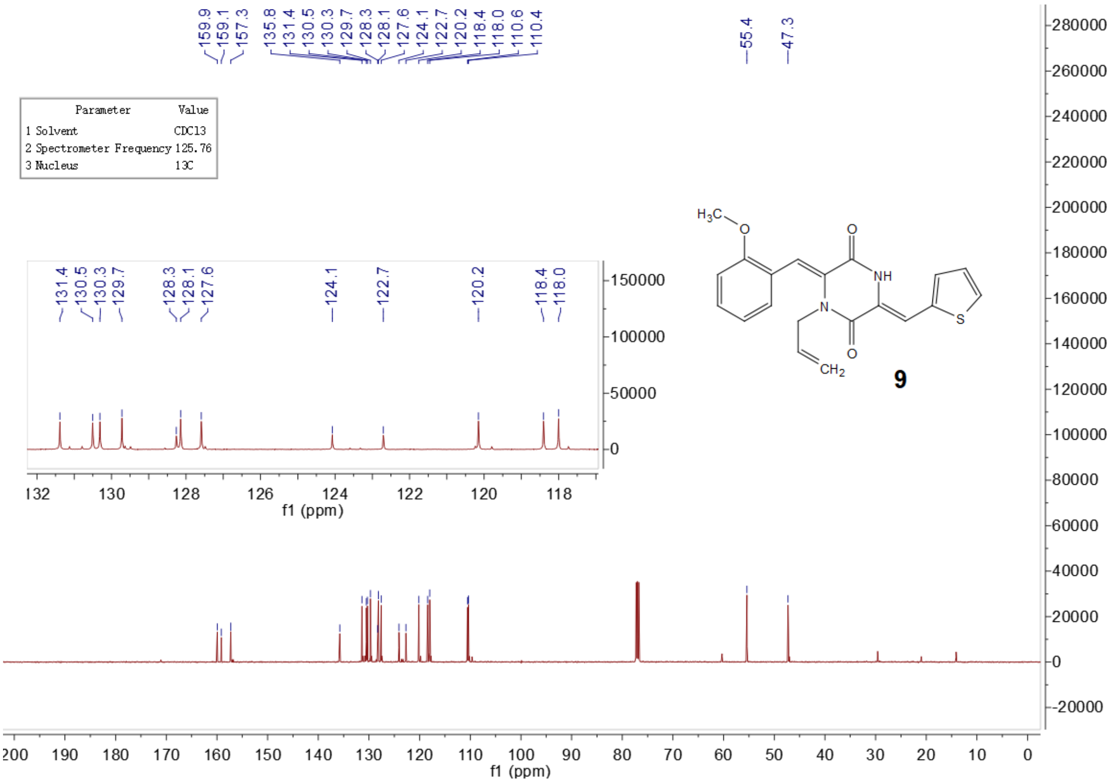

Figure **S15.** ^{1}H NMR of **10.**

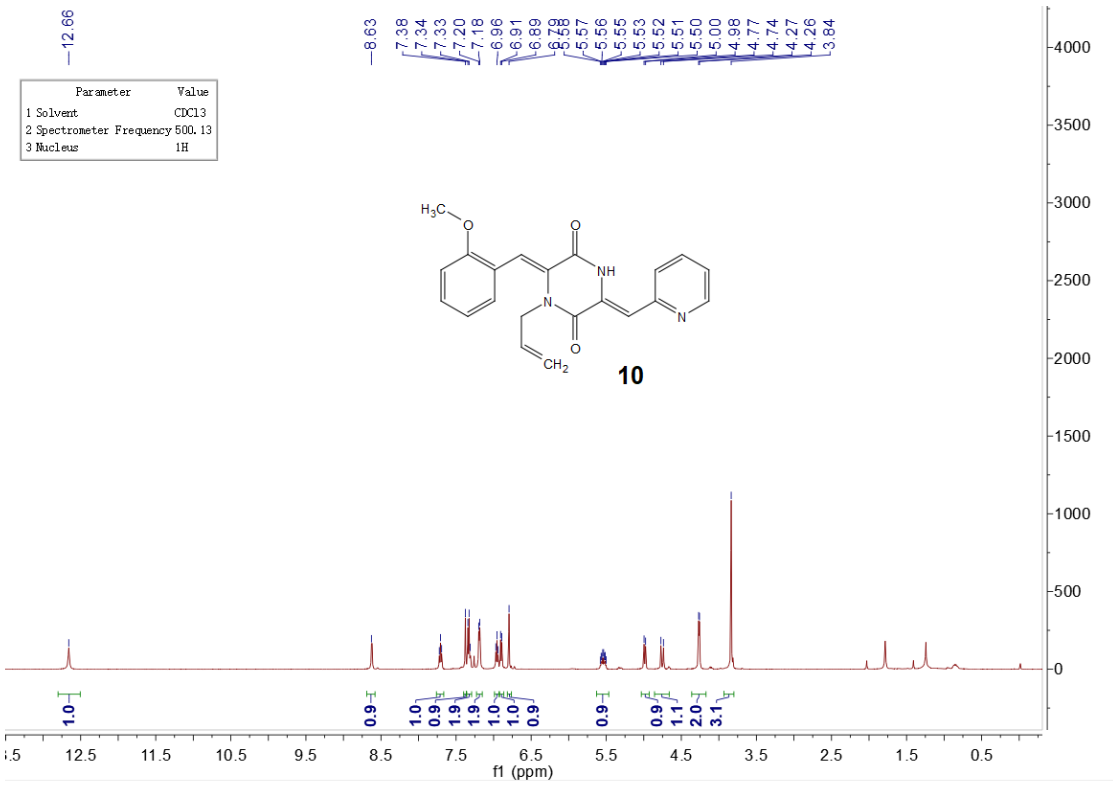

Figure **S16.** ^{13}C NMR of **10.**

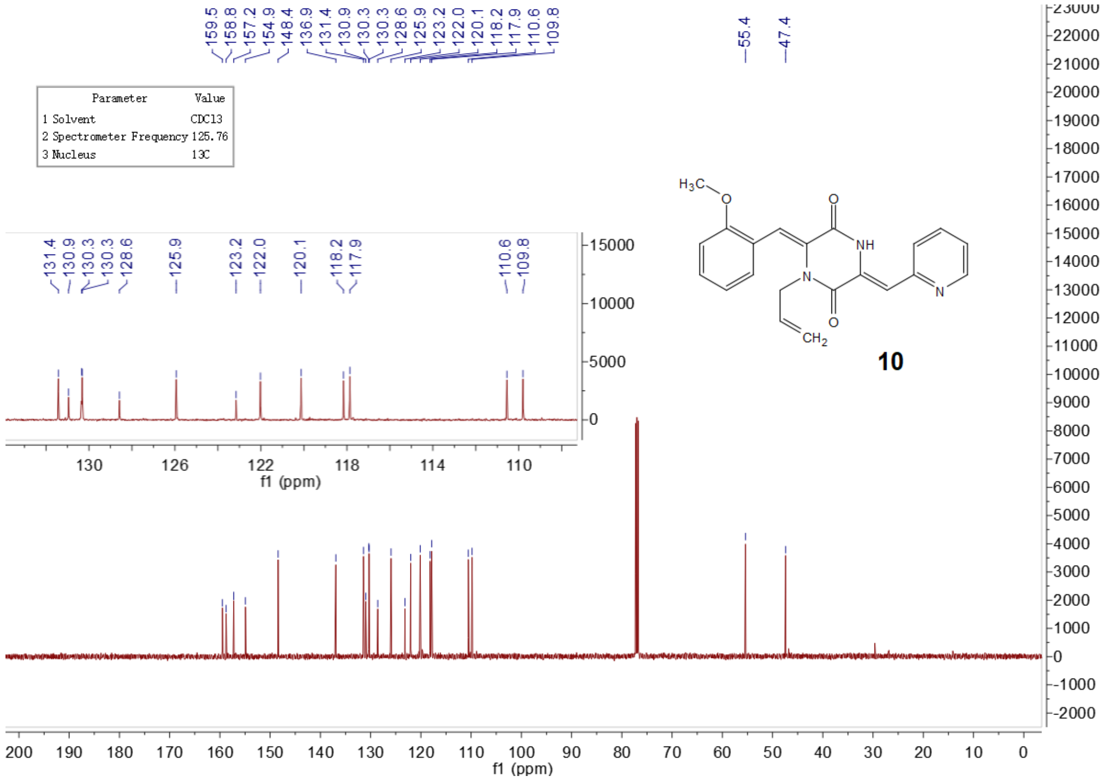

Figure **S17.** ^{1}H NMR of **11.**

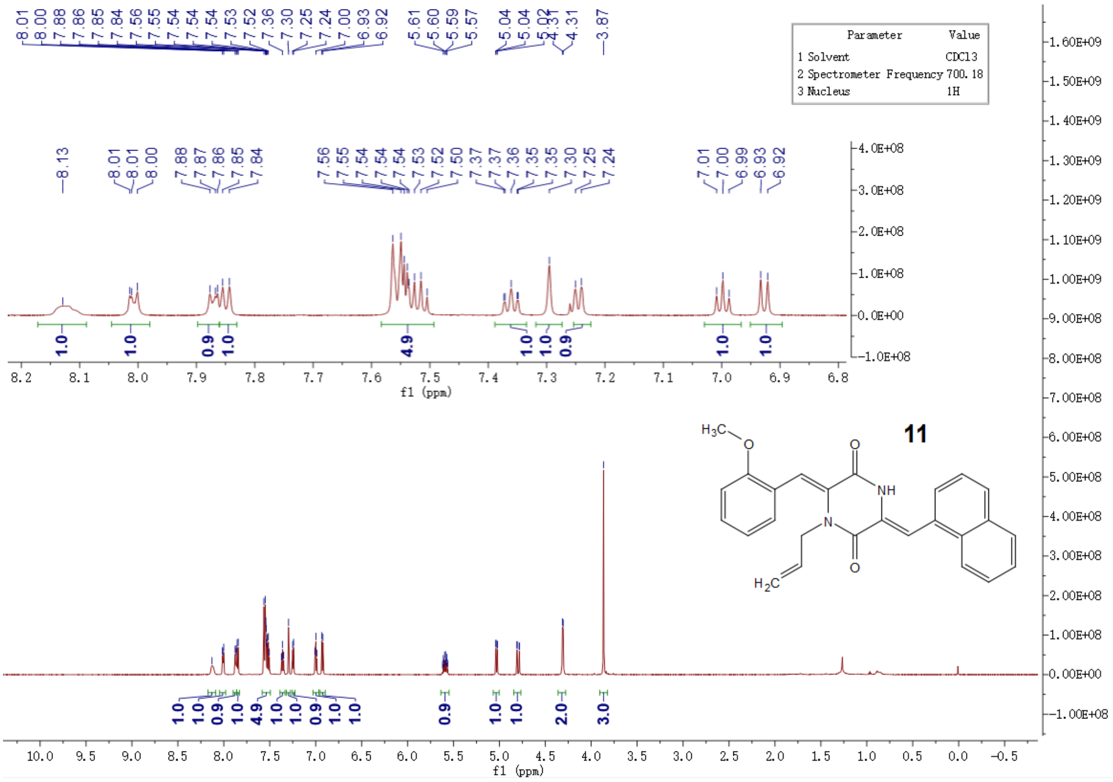

Figure **S18.** ^{13}C NMR of **11.**

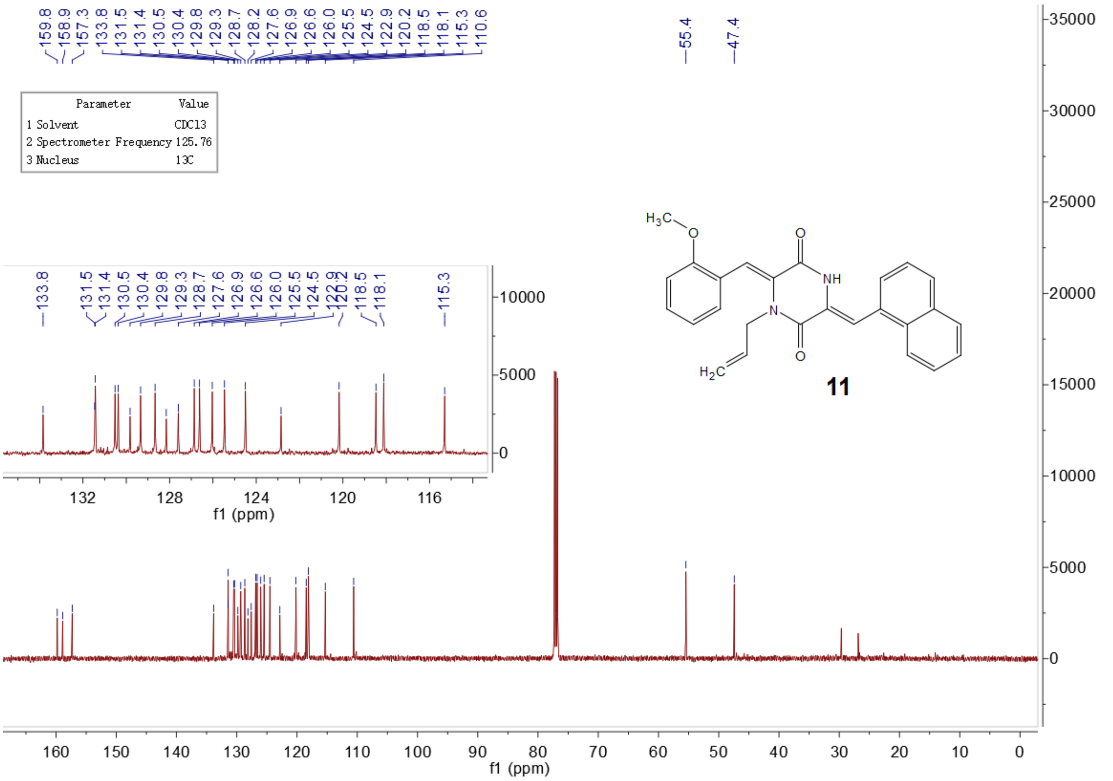

Figure **S19.** ^{1}H NMR of **12.**

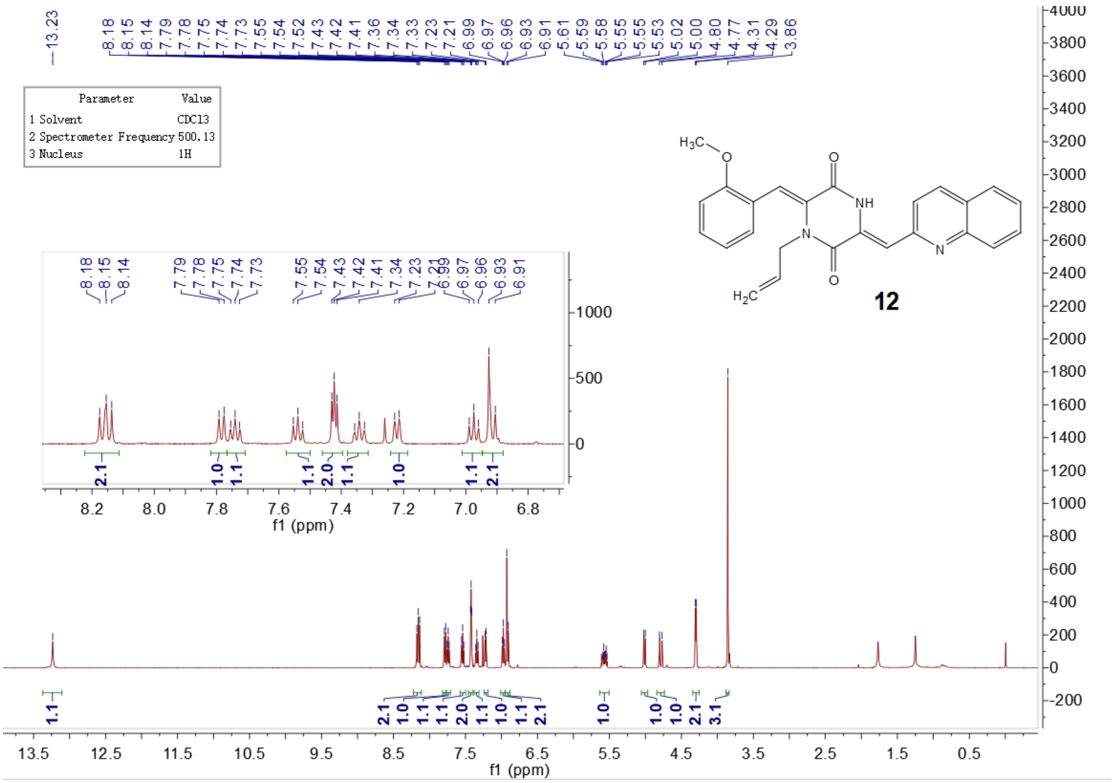

Figure **S20.** ^{13}C NMR of **12.**

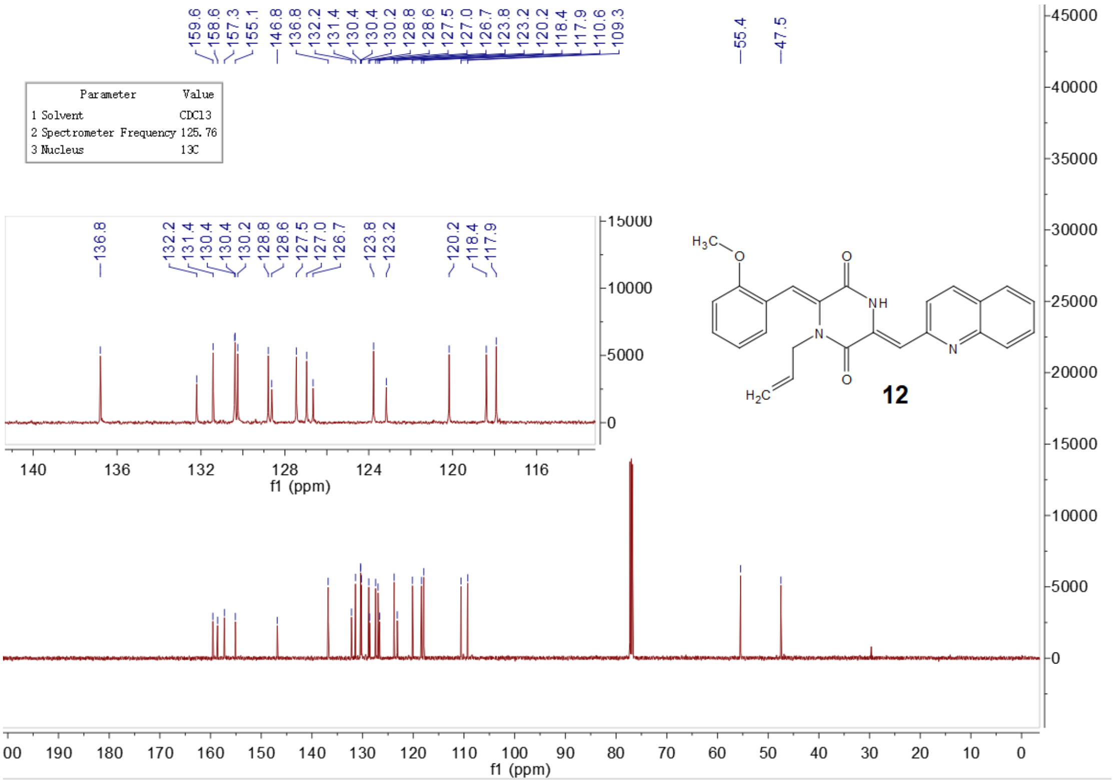

Figure **S21.** ^{1}H NMR of **13.**

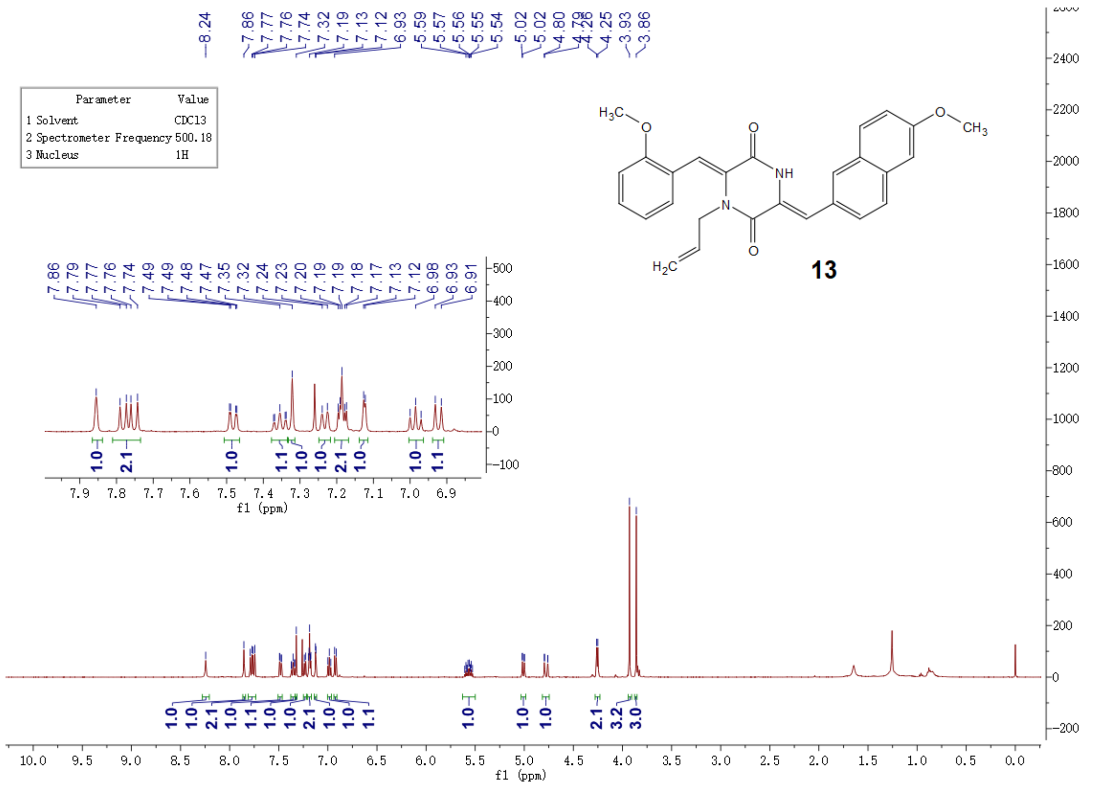

Figure **S22.** ^{13}C NMR of **13.**

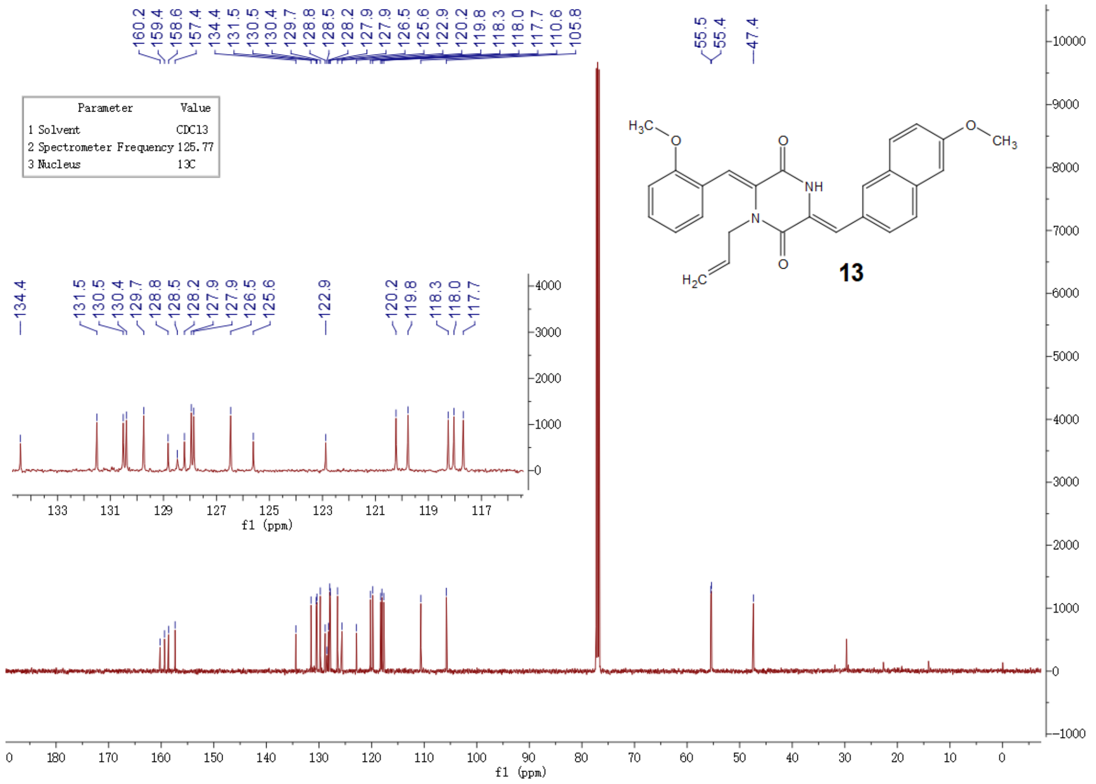

Figure **S23.** ^{1}H NMR of **14.**

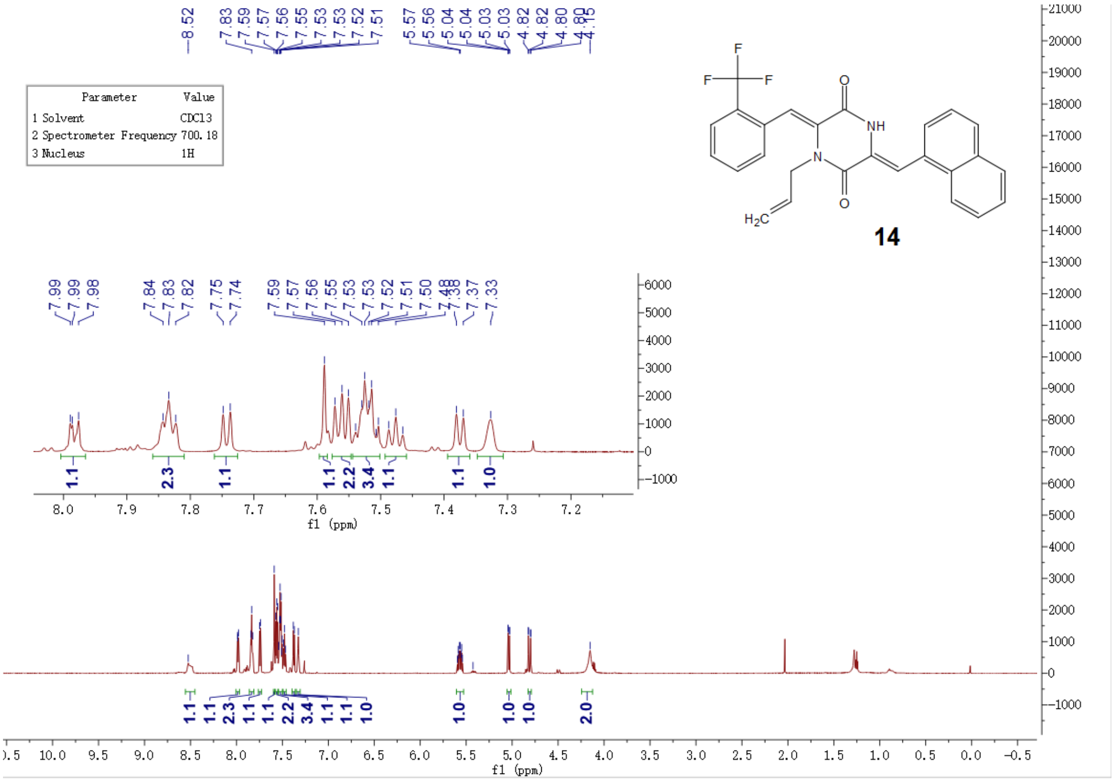

Figure **S24.** ^{13}C NMR of **14.**

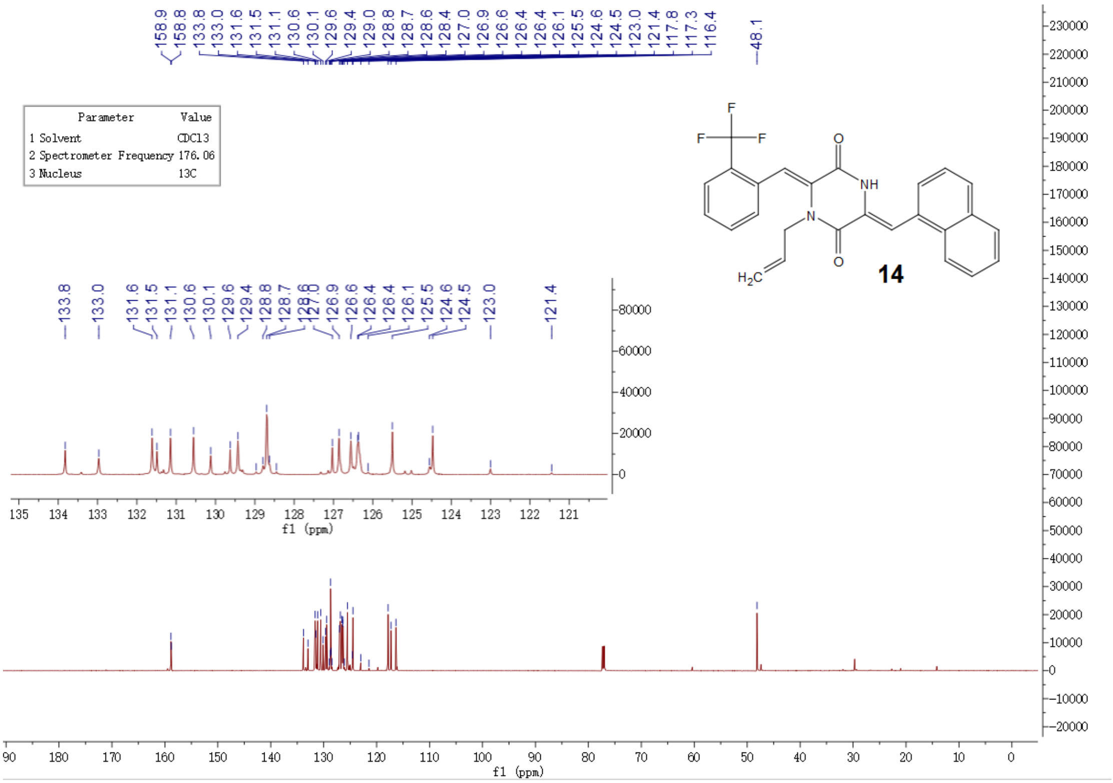

Figure **S25.** ^{1}H NMR of **15.**

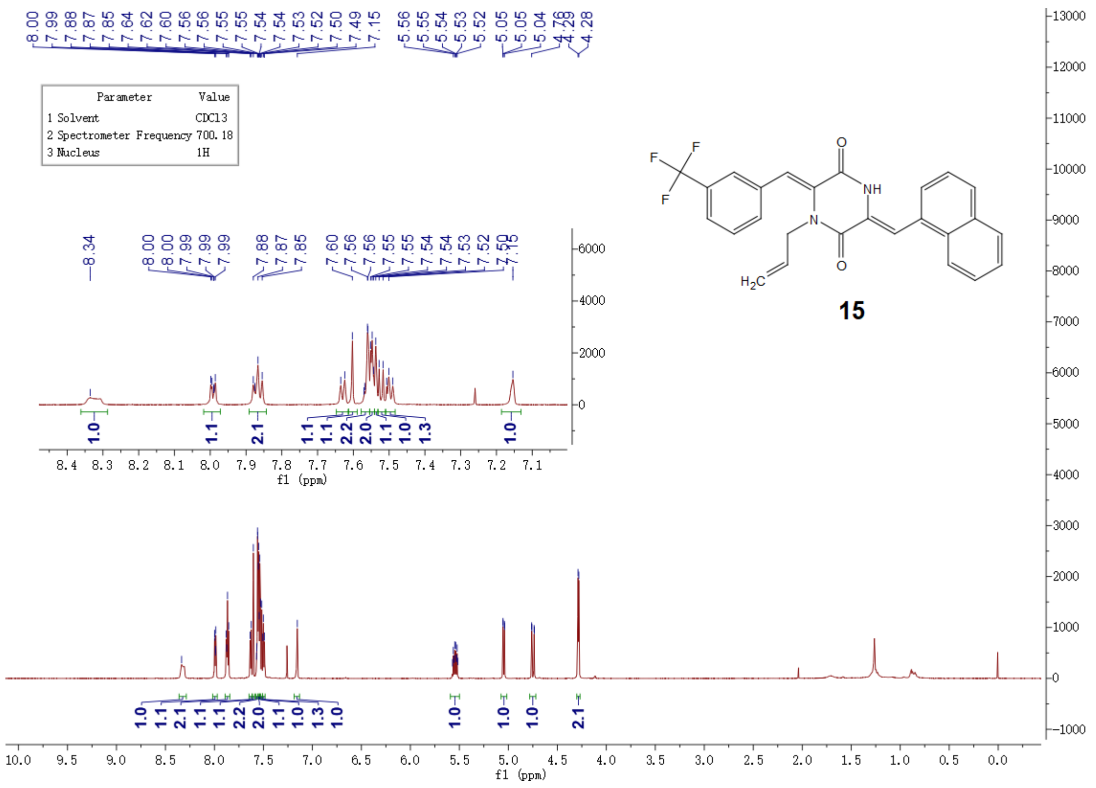

Figure **S26.** ^{13}C NMR of **15.**

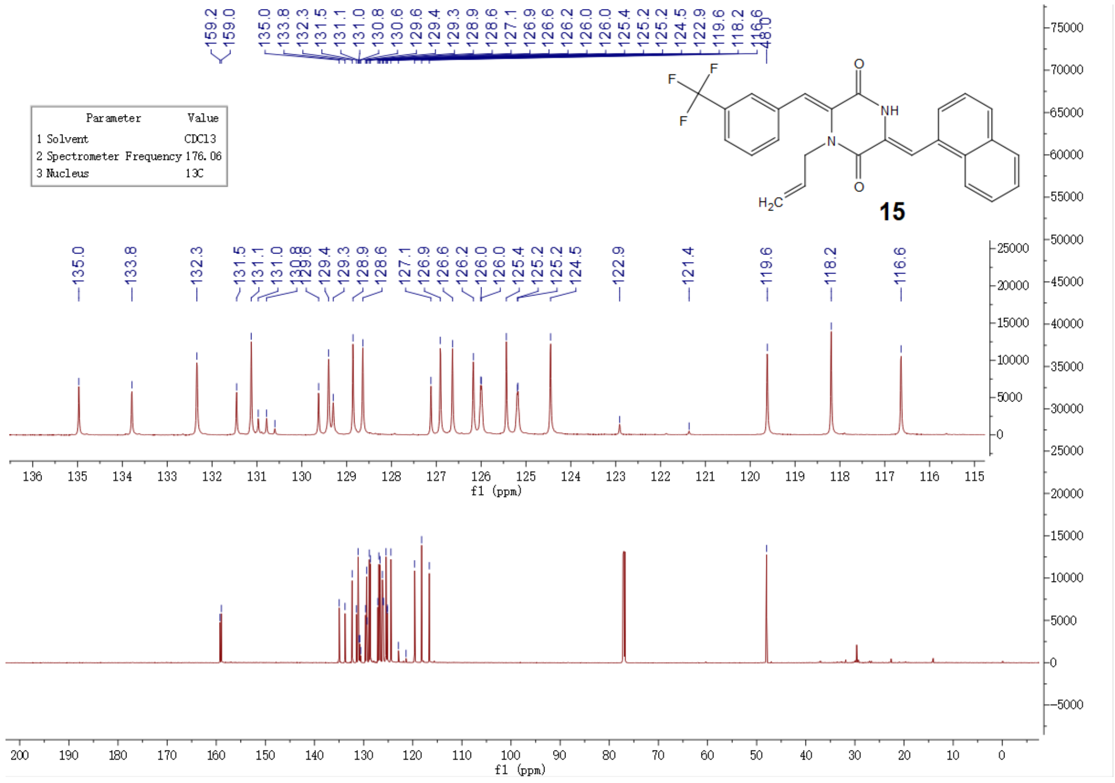

Figure **S27.** ^{1}H NMR of **16.**

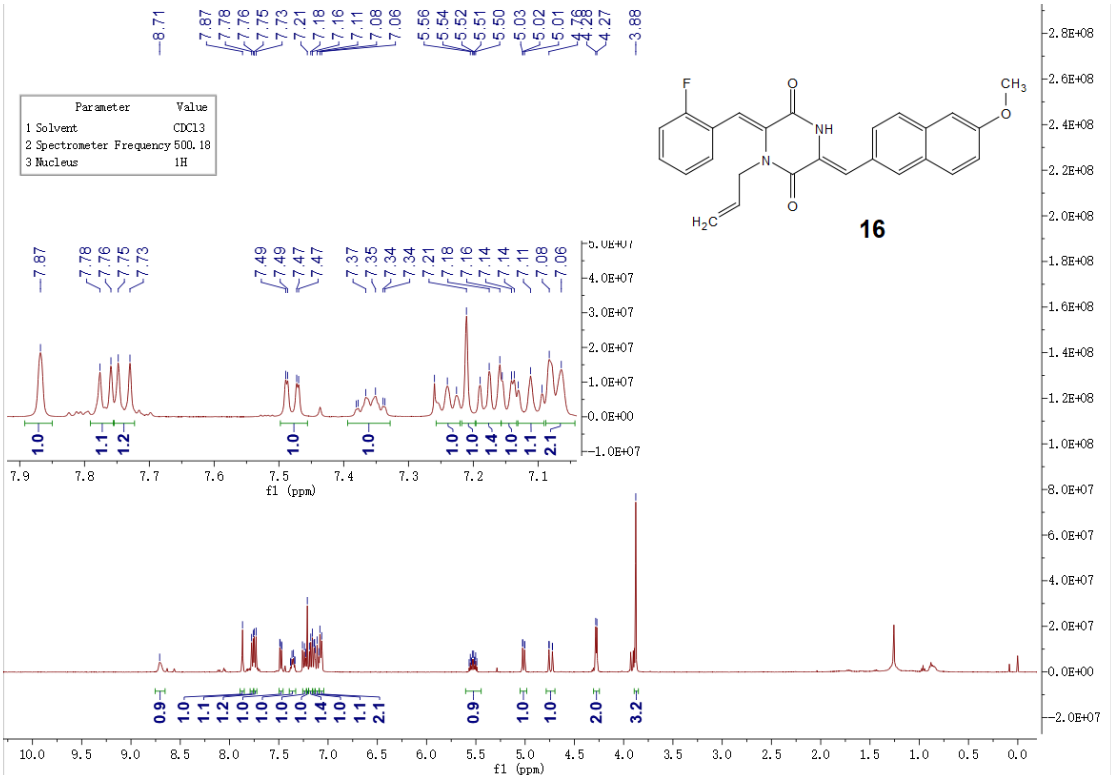

Figure **S28.** ^{13}C NMR of **16.**

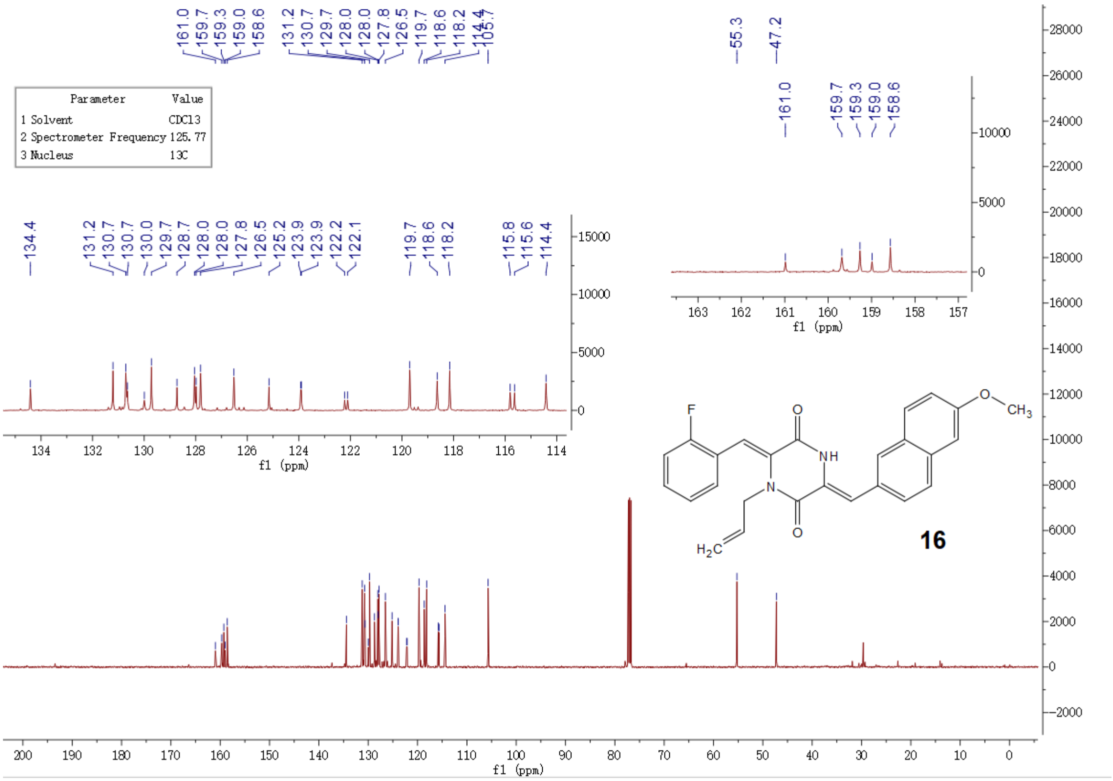

Figure **S29.** ^{1}H-^{1}H NOESY of **11.**

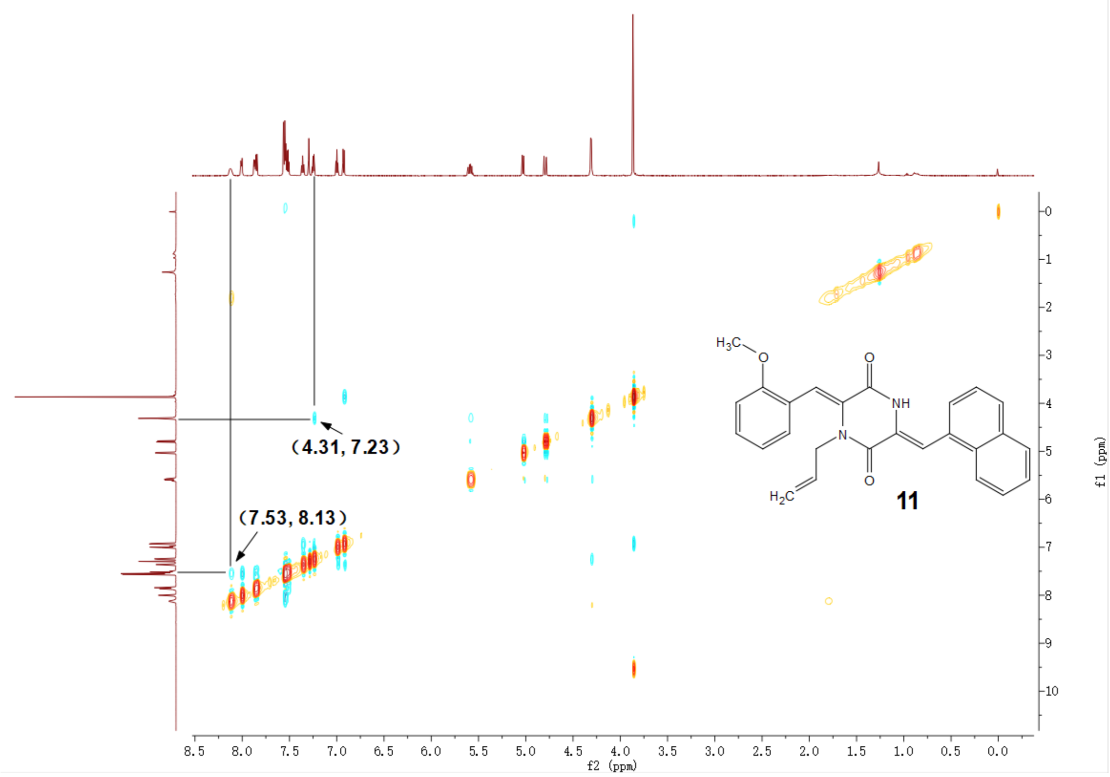

Figure **S30.** HPLC spectrum for compound **1.**

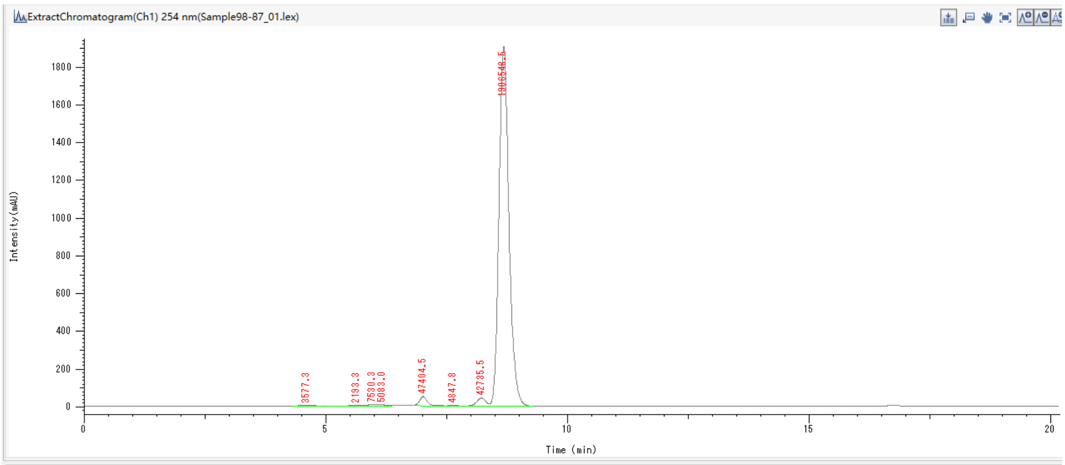

▼ 分析

定量计算设置 | 波形处理设置 | 计算结果 | 样品设置

No.	成分名	RT	面积	面积%	高度	高度%	N	强度	半峰宽	相对RT	校正RT
1	峰 1	4.6	78973.5	0.274	3577.3	0.177	843	6.1	0.37	---	---
2	峰 2	5.6	32716.7	0.114	2193.3	0.109	---	5.5	---	---	---
3	峰 3	6.0	115043.7	0.399	7530.3	0.373	---	11.3	---	---	---
4	峰 4	6.2	51068.0	0.177	5083.0	0.252	---	9.2	---	---	---
5	峰 5	7.0	482879.5	1.677	47404.5	2.347	12193	53.7	0.15	---	---
6	峰 6	7.7	46705.1	0.162	4847.8	0.240	13331	8.0	0.16	---	---
7	峰 7	8.2	558437.7	1.939	42735.5	2.116	8853	45.1	0.21	---	---
8	峰 8	8.7	27434368.6	95.258	1906548.5	94.386	8954	1908.8	0.22	---	---
			28800192.8	100.000	2019920.0	100.000					

Figure **S31.** HPLC spectrum for compound **2.**

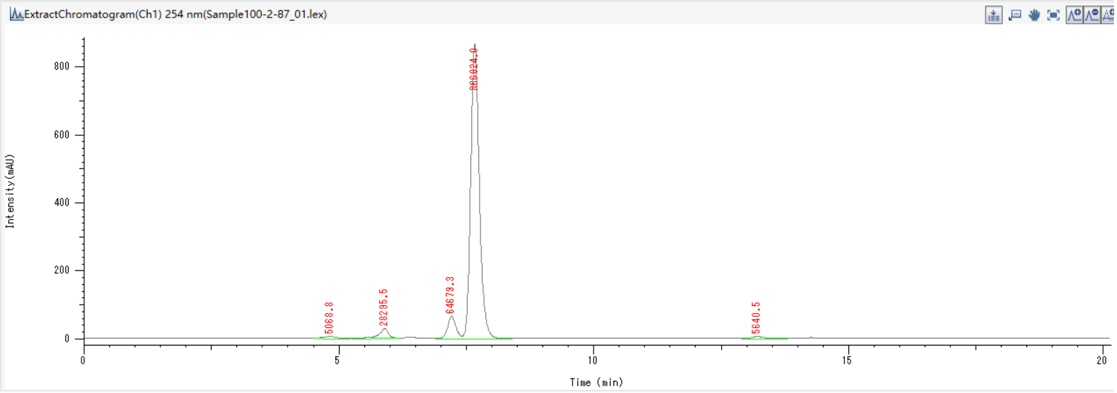

▼ 分析

定量计算设置 | 波形处理设置 | 计算结果 | 样品设置

No.	成分名	RT	面积	面积%	高度	高度%	N	强度	半峰宽	相对RT	校正RT
1	峰 1	4.8	80400.4	0.704	5068.8	0.522	2079	6.2	0.25	---	---
2	峰 2	5.9	351869.8	3.081	28295.5	2.916	7673	29.7	0.16	---	---
3	峰 3	7.2	726121.1	6.358	64679.3	6.664	9807	65.1	0.17	---	---
4	峰 4	7.7	10166326.9	89.022	866824.0	89.317	10517	867.2	0.18	---	---
5	峰 5	13.2	95247.5	0.835	5640.5	0.581	15152	5.7	0.25	---	---
			11419965.8	100.000	970508.0	100.000					

Figure **S32.** HPLC spectrum for compound **3.**

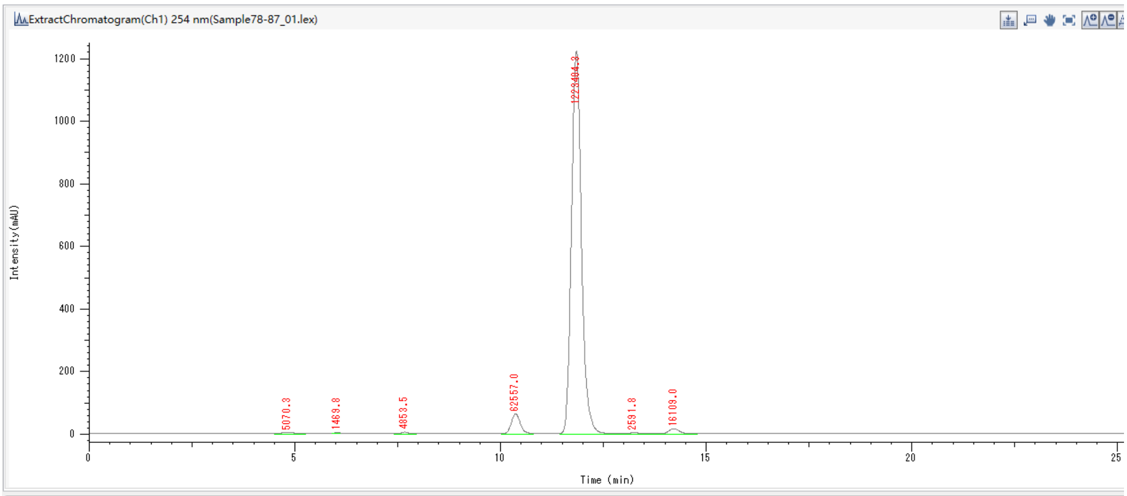

▼ 分析

定量计算设置 | 波形处理设置 | 计算结果 | 样品设置

No.	成分名	RT	面积	面积%	高度	高度%	N	强度	半峰宽	相对RT	校正RT
1	峰 1	4.8	97101.3	0.439	5070.3	0.385	1447	5.2	0.30	---	---
2	峰 2	6.0	8452.9	0.038	1469.8	0.112	26743	4.2	0.09	---	---
3	峰 3	7.7	51190.0	0.231	4853.5	0.369	13763	5.5	0.15	---	---
4	峰 4	10.4	939092.9	4.244	62557.0	4.753	11011	63.2	0.23	---	---
5	峰 5	11.9	20655877.3	93.348	1223484.3	92.960	12030	1224.2	0.25	---	---
6	峰 6	13.2	92554.0	0.418	2591.8	0.197	2467	3.0	0.63	---	---
7	峰 7	14.2	283506.1	1.282	16109.0	1.224	15654	16.4	0.27	---	---
			22127774.5	100.000	1316135.5	100.000					

Figure **S33.** HPLC spectrum for compound **4.**

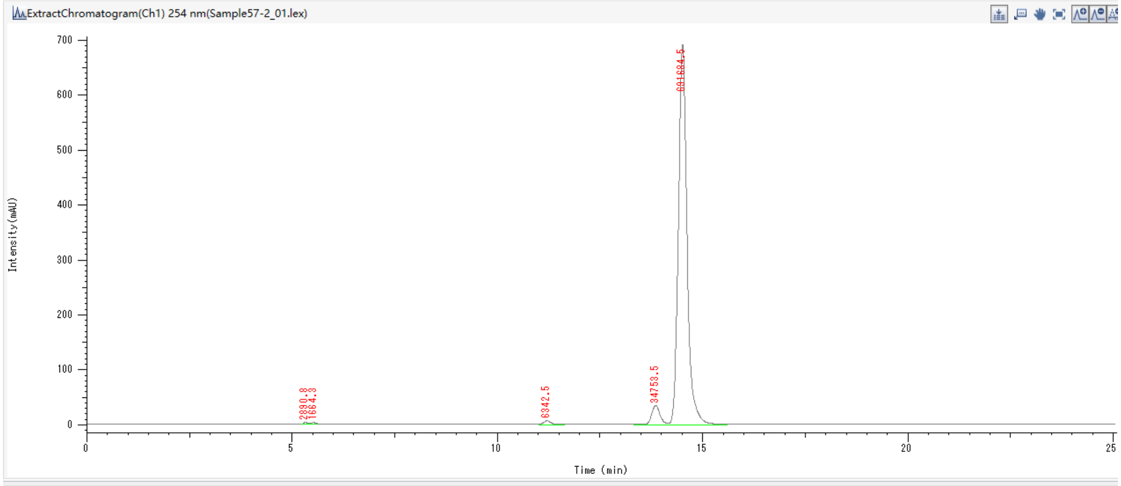

▼ 分析

定量计算设置 | 波形处理设置 | 计算结果 | 样品设置

No.	成分名	RT	面积	面积%	高度	高度%	N	强度	半峰宽	相对RT	校正RT
1	峰 1	5.3	15214.3	0.142	2890.8	0.392	21632	3.8	0.09	---	---
2	峰 2	5.5	9829.6	0.092	1664.3	0.226	22054	3.4	0.09	---	---
3	峰 3	11.2	72957.5	0.682	6342.5	0.860	23777	6.5	0.17	---	---
4	峰 4	13.9	478310.8	4.470	34753.5	4.713	25046	34.7	0.21	---	---
5	峰 5	14.5	10123097.0	94.614	691684.5	93.809	25240	691.6	0.22	---	---
			10699409.2	100.000	737335.5	100.000					

Figure **S34.** HPLC spectrum for compound **5.**

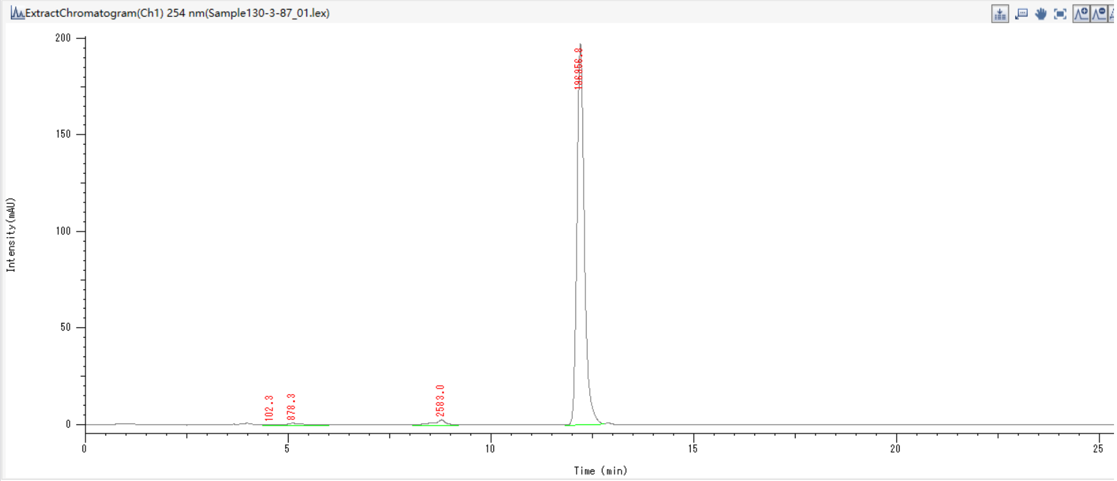

▼ 分析

定量计算设置 | 波形处理设置 | 计算结果 | 样品设置

No.	成分名	RT	面积	面积%	高度	高度%	N	强度	半峰宽	相对RT	校正RT
1	峰 1	4.6	1578.8	0.062	102.3	0.051	10751	-0.3	0.10	---	---
2	峰 2	5.1	20935.1	0.816	878.3	0.438	1290	0.5	0.34	---	---
3	峰 3	8.8	49187.0	1.917	2583.0	1.288	11761	2.2	0.19	---	---
4	峰 4	12.2	2494181.4	97.205	196956.8	98.223	23606	196.8	0.19	---	---
			2565882.3	100.000	200520.3	100.000					

Figure **S35.** HPLC spectrum for compound **6.**

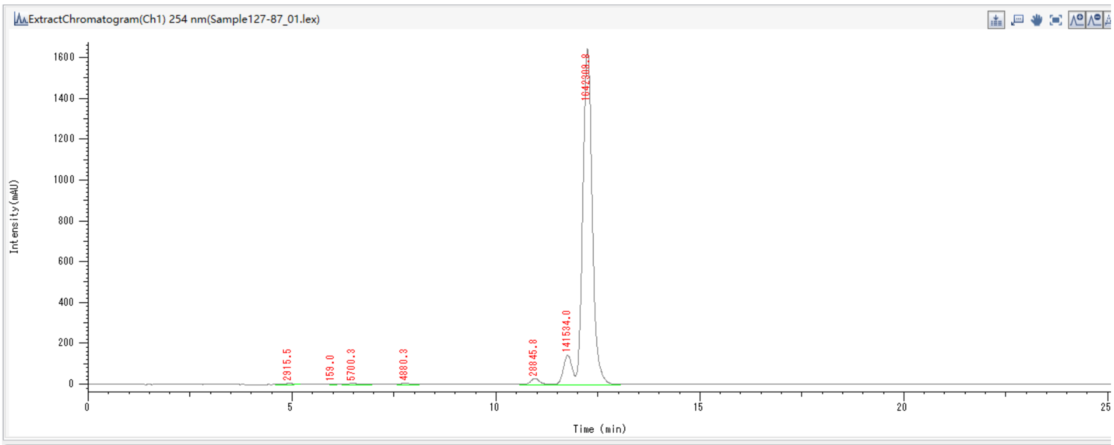

分析

定量计算设置 | 波形处理设置 | 计算结果 | 样品设置

No.	成分名	RT	面积	面积%	高度	高度%	N	强度	半峰宽	相对RT	校正RT
1	峰 1	4.9	59480.4	0.206	2915.5	0.160	924	0.4	0.38	---	---
2	峰 2	6.0	1024.6	0.004	159.0	0.009	---	-2.5	---	---	---
3	峰 3	6.5	71206.6	0.246	5700.3	0.312	6902	2.4	0.18	---	---
4	峰 4	7.8	69330.6	0.240	4880.3	0.267	6304	1.6	0.23	---	---
5	峰 5	11.0	431857.6	1.493	28845.8	1.579	12866	25.4	0.23	---	---
6	峰 6	11.8	2069538.1	7.153	141534.0	7.750	13496	138.2	0.24	---	---
7	峰 7	12.3	26231745.1	90.658	1642309.8	89.923	14201	1639.0	0.24	---	---
			28934182.9	100.000	1826344.5	100.000					

Figure **S36.** HPLC spectrum for compound **7.**

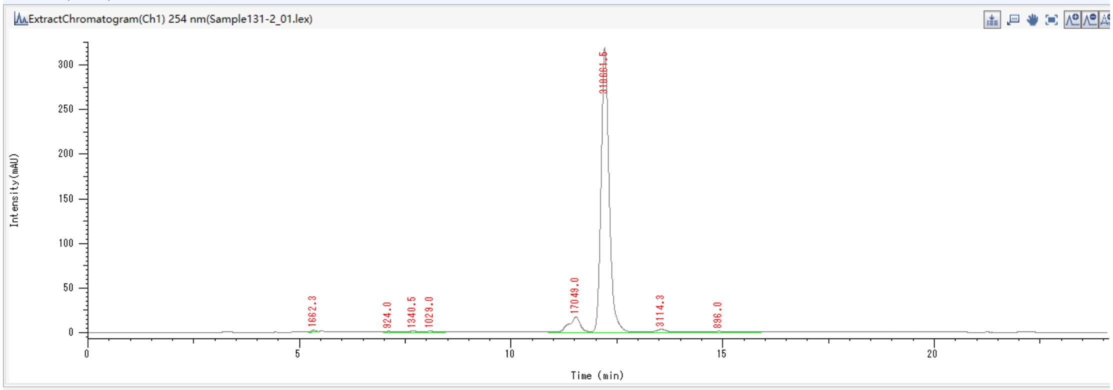

分析

定量计算设置 | 波形处理设置 | 计算结果 | 样品设置

No.	成分名	RT	面积	面积%	高度	高度%	N	强度	半峰宽	相对RT	校正RT
1	峰 1	5.4	9554.9	0.205	1662.3	0.482	19257	2.3	0.09	---	---
2	峰 2	7.1	12783.8	0.274	924.0	0.268	9490	1.1	0.17	---	---
3	峰 3	7.7	13121.9	0.281	1340.5	0.389	16404	1.5	0.14	---	---
4	峰 4	8.1	12360.4	0.265	1029.0	0.299	14137	1.2	0.16	---	---
5	峰 5	11.5	309529.3	6.631	17049.0	4.946	7993	17.1	0.30	---	---
6	峰 6	12.2	4238815.2	90.807	318661.5	92.452	21232	318.7	0.20	---	---
7	峰 7	13.6	51773.5	1.109	3114.3	0.904	19824	3.3	0.23	---	---
8	峰 8	14.9	19999.3	0.428	896.0	0.260	13256	1.1	0.31	---	---
			4667938.2	100.000	344676.5	100.000					

Figure **S37.** HPLC spectrum for compound **8.**

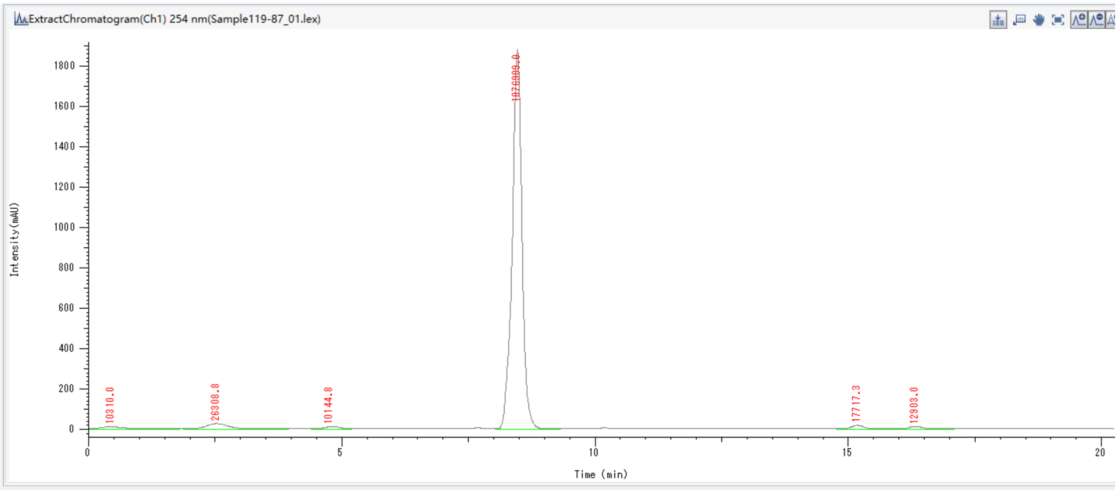

▼ 分析

定量计算设置 | 波形处理设置 | 计算结果 | 样品设置

No.	成分名	RT	面积	面积%	高度	高度%	N	强度	半峰宽	相对RT	校正RT
1	峰 1	0.5	328674.4	1.208	10310.0	0.528	6	10.7	0.44	---	---
2	峰 2	2.5	809696.4	2.975	26308.8	1.346	171	26.3	0.46	---	---
3	峰 3	4.8	204106.6	0.750	10144.8	0.519	1119	10.8	0.34	---	---
4	峰 4	8.5	25331753.9	93.076	1876989.0	96.040	10632	1877.6	0.19	---	---
5	峰 5	15.2	305898.0	1.124	17717.3	0.907	19340	17.7	0.26	---	---
6	峰 6	16.3	236051.5	0.867	12903.0	0.660	19952	12.9	0.27	---	---
			27216180.8	100.000	1954372.8	100.000					

Figure **S38.** HPLC spectrum for compound **9.**

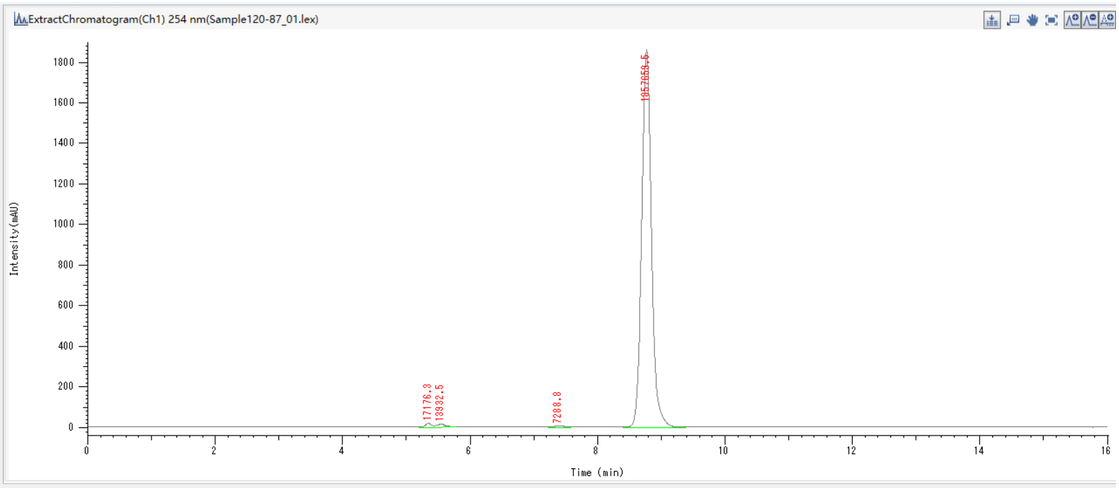

▼ 分析

定量计算设置 | 波形处理设置 | 计算结果 | 样品设置

No.	成分名	RT	面积	面积%	高度	高度%	N	强度	半峰宽	相对RT	校正RT
1	峰 1	5.4	104342.1	0.524	17176.3	0.906	17055	18.4	0.10	---	---
2	峰 2	5.6	120334.1	0.605	13932.5	0.735	9555	16.0	0.13	---	---
3	峰 3	7.4	62024.8	0.312	7288.8	0.384	16112	7.9	0.14	---	---
4	峰 4	8.8	19613512.7	98.559	1857659.5	97.975	17564	1858.1	0.16	---	---
			19900213.7	100.000	1896057.0	100.000					

Figure **S39.** HPLC spectrum for compound **10.**

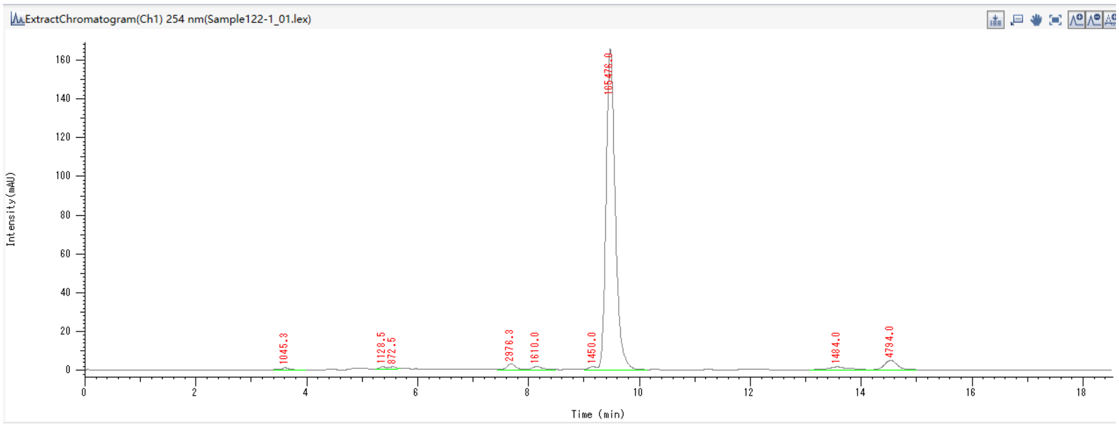

▼ 分析

定量计算设置 | 波形处理设置 | 计算结果 | 样品设置

No.	成分名	RT	面积	面积%	高度	高度%	N	强度	半峰宽	相对RT	校正RT
1	峰 1	3.6	8366.1	0.399	1045.3	0.578	5770	1.1	0.11	---	---
2	峰 2	5.4	7678.7	0.366	1128.5	0.624	12194	1.6	0.11	---	---
3	峰 3	5.6	6870.5	0.328	872.5	0.482	10369	1.6	0.13	---	---
4	峰 4	7.7	29916.1	1.427	2976.3	1.646	14668	3.1	0.15	---	---
5	峰 5	8.2	19233.4	0.917	1610.0	0.890	12148	1.7	0.17	---	---
6	峰 6	9.2	12434.8	0.593	1450.0	0.802	---	1.6	---	---	---
7	峰 7	9.5	1900743.9	90.664	165476.0	91.506	16856	165.6	0.17	---	---
8	峰 8	13.6	36183.4	1.726	1484.0	0.821	10829	1.5	0.31	---	---
9	峰 9	14.5	75047.1	3.580	4794.0	2.651	20464	4.9	0.24	---	---
			2096473.9	100.000	180836.5	100.000					

Figure **S40.** HPLC spectrum for compound **11.**

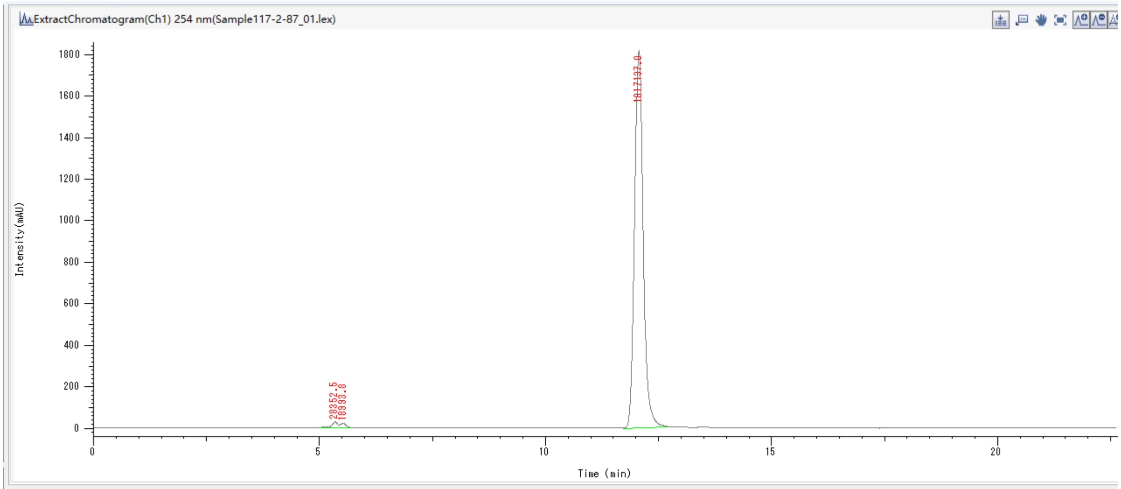

▼ 分析

定量计算设置 | 波形处理设置 | 计算结果 | 样品设置

No.	成分名	RT	面积	面积%	高度	高度%	N	强度	半峰宽	相对RT	校正RT
1	峰 1	5.4	220824.4	0.897	28352.5	1.521	12170	30.8	0.11	---	---
2	峰 2	5.5	166865.1	0.678	18993.8	1.019	---	21.6	---	---	---
3	峰 3	12.1	24228352.8	98.425	1817137.0	97.460	20138	1819.4	0.20	---	---
			24616042.3	100.000	1864483.3	100.000					

Figure **S41.** HPLC spectrum for compound **12.**

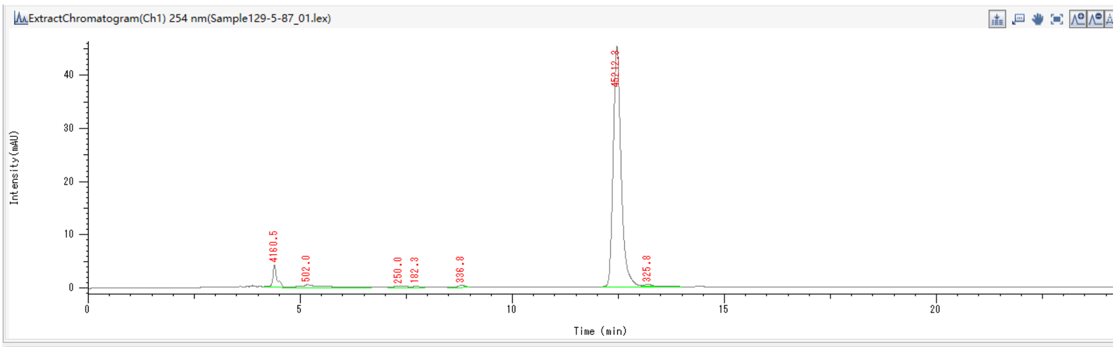

▼ 分析

定量计算设置 | 波形处理设置 | 计算结果 | 样品设置

No.	成分名	RT	面积	面积%	高度	高度%	N	强度	半峰宽	相对RT	校正RT
1	峰 1	4.4	28275.5	4.343	4160.5	8.163	16622	4.3	0.08	---	---
2	峰 2	5.2	15875.4	2.438	502.0	0.985	1097	0.6	0.37	---	---
3	峰 3	7.3	4812.5	0.739	250.0	0.490	2722	0.3	0.33	---	---
4	峰 4	7.7	1832.6	0.281	182.3	0.358	10203	0.3	0.18	---	---
5	峰 5	8.8	3487.5	0.536	336.8	0.661	16299	0.4	0.16	---	---
6	峰 6	12.5	593427.3	91.138	45212.3	88.705	23508	45.4	0.19	---	---
7	峰 7	13.2	3422.1	0.525	325.8	0.638	36398	0.6	0.16	---	---
			651132.8	100.000	50969.5	100.000					

Figure **S42.** HPLC spectrum for compound **13.**

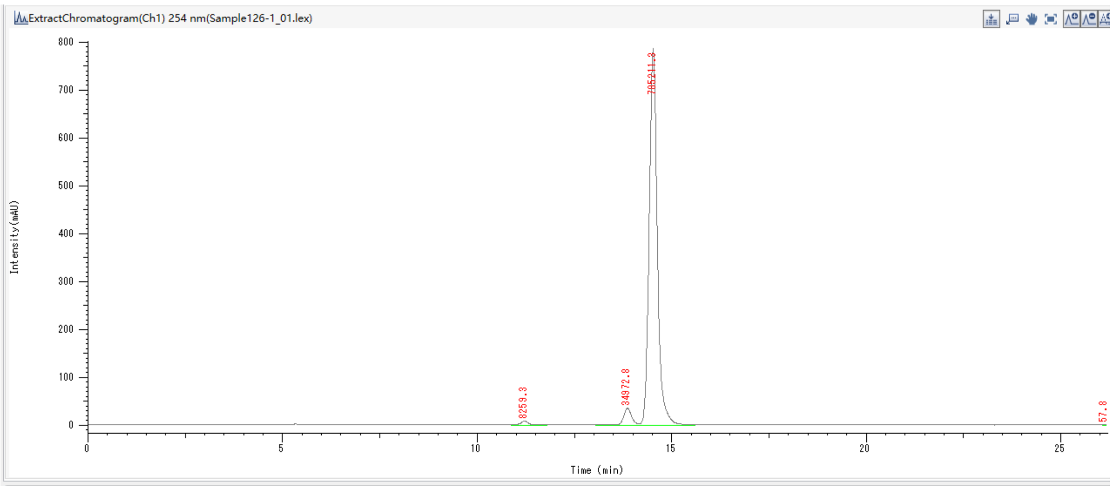

▼ 分析

定量计算设置 | 波形处理设置 | 计算结果 | 样品设置

No.	成分名	RT	面积	面积%	高度	高度%	N	强度	半峰宽	相对RT	校正RT
1	峰 1	11.2	105842.6	0.880	8259.3	0.997	21210	8.0	0.18	---	---
2	峰 2	13.9	484210.7	4.026	34972.8	4.221	24713	34.8	0.21	---	---
3	峰 3	14.5	11437859.3	95.092	785211.3	94.775	25393	785.0	0.21	---	---
4	峰 4	26.1	275.5	0.002	57.8	0.007	637039	-0.3	0.08	---	---
			12028188.0	100.000	828501.0	100.000					

Figure **S43.** HPLC spectrum for compound **14.**

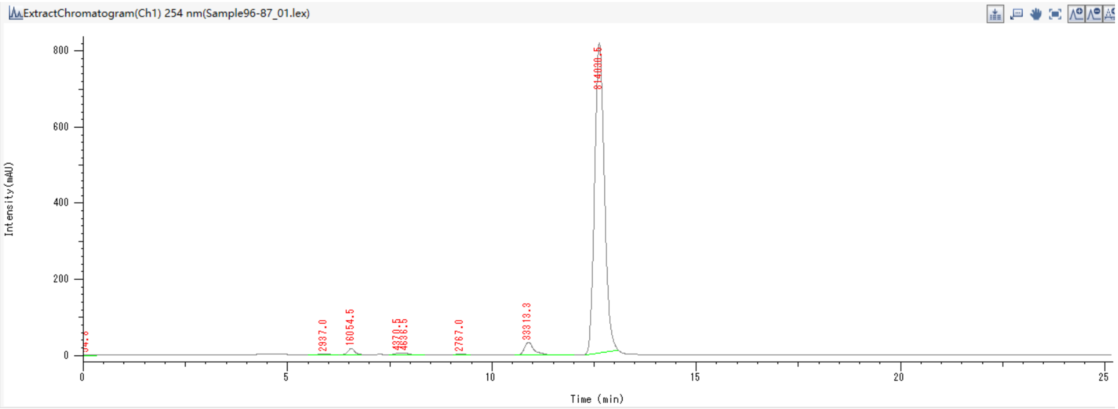

▼ 分析

定量计算设置 | 波形处理设置 | 计算结果 | 样品设置

No.	成分名	RT	面积	面积%	高度	高度%	N	强度	半峰宽	相对RT	校正RT
1	峰 1	0.1	399.4	0.003	54.8	0.006	8	0.1	0.06	---	---
2	峰 2	5.9	41299.6	0.287	2937.0	0.334	4345	3.5	0.21	---	---
3	峰 3	6.6	172033.0	1.197	16054.5	1.828	9043	17.3	0.16	---	---
4	峰 4	7.7	41061.8	0.286	4370.5	0.498	---	4.9	---	---	---
5	峰 5	7.9	49627.2	0.345	4636.5	0.528	---	5.1	---	---	---
6	峰 6	9.2	32147.2	0.224	2767.0	0.315	13077	3.4	0.19	---	---
7	峰 7	10.9	541605.4	3.769	33313.3	3.794	13336	33.5	0.22	---	---
8	峰 8	12.6	13492734.3	93.889	814030.5	92.697	13471	820.7	0.26	---	---
			14370907.9	100.000	878164.0	100.000					

Figure **S44.** HPLC spectrum for compound **15.**

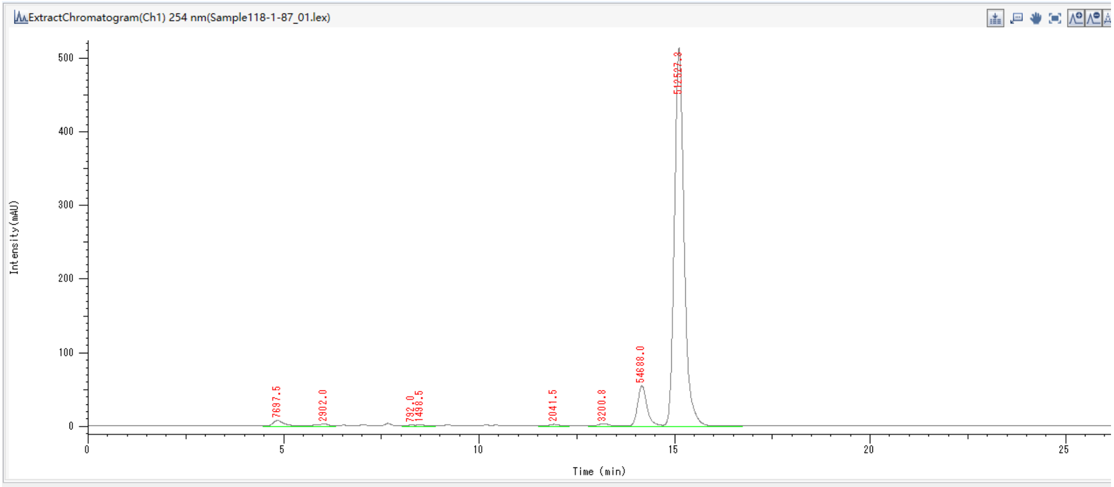

▼ 分析

定量计算设置 | 波形处理设置 | 计算结果 | 样品设置

No.	成分名	RT	面积	面积%	高度	高度%	N	强度	半峰宽	相对RT	校正RT
1	峰 1	4.9	148980.9	1.491	7697.5	1.315	1791	7.8	0.27	---	---
2	峰 2	6.0	46139.5	0.462	2902.0	0.496	5539	3.3	0.19	---	---
3	峰 3	8.3	9821.1	0.098	792.0	0.135	---	1.2	---	---	---
4	峰 4	8.5	20558.7	0.206	1498.5	0.256	12449	1.9	0.18	---	---
5	峰 5	11.9	31910.0	0.319	2041.5	0.349	14530	2.3	0.23	---	---
6	峰 6	13.2	56491.1	0.565	3200.8	0.547	13483	3.4	0.27	---	---
7	峰 7	14.2	904418.5	9.050	54688.0	9.343	18251	54.9	0.25	---	---
8	峰 8	15.1	8774965.4	87.809	512527.3	87.559	19587	512.7	0.25	---	---
			9993285.2	100.000	585347.5	100.000					

Figure **S45.** HPLC spectrum for compound **16.**

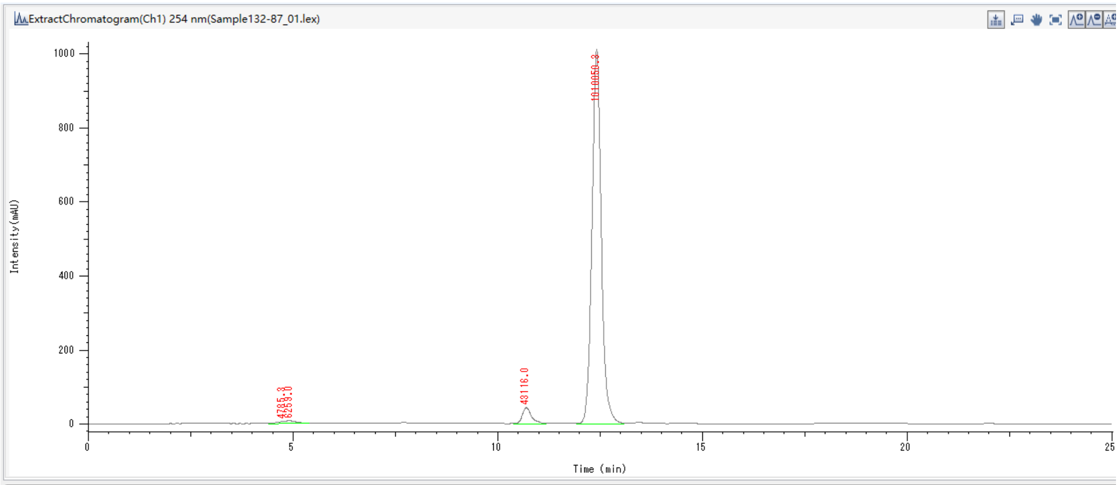

▼ 分析

定量计算设置 | 波形处理设置 | 计算结果 | 样品设置

No.	成分名	RT	面积	面积%	高度	高度%	N	强度	半峰宽	相对RT	校正RT
1	峰 1	4.8	47192.0	0.280	4785.3	0.450	---	6.1	---	---	---
2	峰 2	4.9	112770.3	0.670	6259.0	0.588	---	7.6	---	---	---
3	峰 3	10.7	644217.1	3.827	43116.0	4.051	13418	43.5	0.22	---	---
4	峰 4	12.4	16028294.1	95.223	1010050.3	94.911	15282	1010.7	0.24	---	---
			16832473.5	100.000	1064210.5	100.000					

References

1. Liao, S. R.; Du, L. J.; Qin, X. C.; Xu, L.; Wang, J. F.; Zhou, X. F.; Tu, Z. C.; Li, J.; Liu, Y. H., *Tetrahedron* **2016,** 72, (8), 1051-1057.

2. Liao, S. R.; Xu, Y.; Tang, Y.; Wang, J. F.; Zhou, X. F.; Xu, L.; Liu, Y. H., *Rsc Adv* **2015,** 5, (63), 51020-51026.
